# Assessment of Immunological Response and Impacts on Fertility Following Intrauterine Vaccination Delivered to Swine in an Artificial Insemination Dose

**DOI:** 10.3389/fimmu.2020.01015

**Published:** 2020-05-27

**Authors:** Glenn Hamonic, J. Alex Pasternak, Siew Hon Ng, Kezia R. Fourie, Olena M. Simko, Brodie Deluco, Heather L. Wilson

**Affiliations:** ^1^Department of Agricultural, Food and Nutritional Science, University of Alberta, Edmonton, AB, Canada; ^2^Large Animal Clinical Sciences, Western College of Veterinary Medicine, University of Saskatchewan, Saskatoon, SK, Canada; ^3^Department of Animal Science, Purdue University, West Lafayette, IN, United States; ^4^Vaccine and Infectious Disease Organization (VIDO)-International Vaccine Centre (InterVac), University of Saskatchewan, Saskatoon, SK, Canada; ^5^Department of Veterinary Microbiology, Western College of Veterinary Medicine, University of Saskatchewan, Saskatoon, SK, Canada

**Keywords:** breeding, pigs, estrus, mucosal vaccine, uterus, semen, adjuvant

## Abstract

To protect the health of sows and gilts, significant investments are directed toward the development of vaccines against infectious agents that impact reproduction. We developed an intrauterine vaccine that can be delivered with semen during artificial insemination to induce mucosal immunity in the reproductive tract. An *in vitro* culture of uterine epithelial cells was used to select an adjuvant combination capable of recruiting antigen-presenting cells into the uterus. Adjuvant polyinosinic:polycytidylic acid (poly I:C), alone or in combination, induced expression of interferon gamma, tumor necrosis factor alpha, and select chemokines. A combination adjuvant consisting of poly I:C, host defense peptide and polyphosphazene (Triple Adjuvant; TriAdj), which previously was shown to induce robust mucosal and systemic humoral immunity when administered to the uterus in rabbits, was combined with boar semen to evaluate changes in localized gene expression and cellular recruitment, *in vivo*. Sows bred with semen plus TriAdj had decreased γδ T cells and monocytes in blood, however, no corresponding increase in the number of monocytes and macrophages was detected in the endometrium. Compared to sows bred with semen alone, sows bred with semen plus TriAdj showed increased CCL2 gene expression in the epithelial layer. These data suggest that the adjuvants may further augment a local immune response and, therefore, may be suitable for use in an intrauterine vaccine. When inactivated porcine parvovirus (PPV) formulated with the TriAdj was administered to the pig uterus during estrus along with semen, we observed induction of PPV antibodies in serum but only when the pigs were already primed with parenteral PPV vaccines. Recombinant protein vaccines and inactivated PPV vaccines administered to the pig uterus during breeding as a primary vaccine alone failed to induce significant humoral immunity. More trials need to be performed to clarify whether repeated intrauterine vaccination can trigger strong humoral immunity or whether the primary vaccine needs to be administered via a systemic route to promote a mucosal and systemic immune response.

## Introduction

Mucosal vaccination of livestock has the potential for several benefits over classical parenteral vaccinations, including the initiation of a strong mucosal and systemic immune response (1, 2) while reducing the incidence of common needle-stick injuries by veterinarians (3). However, several challenges need to be overcome in order to generate a successful mucosal immune response including avoiding vaccine elimination by the flow of mucosal fluids across mucosal surfaces, recruitment of antigen presenting cells (APCs), and targeting of the vaccine toward APCs (4). Mucosal surfaces are primed to induce a tolerogenic response toward antigens thereby limiting the reaction to microflora, food, and environmental particles (5). Currently, no studies have identified a commensal flora in the upper reproductive tract of pigs, which may mean that the porcine uterus may be less predisposed to a tolerogenic bias to antigens encountered at its surface. In fact, studies in rats and rabbits have shown that the uterus may be a suitable immunization site as vaccines delivered to the uterus triggered a measurable antigen-specific systemic and local humoral immunity (6–8). Because a number of economically important diseases in pigs such as porcine parvovirus (PPV) and porcine reproductive and respiratory syndrome virus (PRRSV) impact reproduction, it may be very beneficial to have a mode of vaccine delivery that triggers a strong mucosal immune response in the uterus to protect growing fetuses (9). For livestock systems that use natural breeding, the uterus is not readily accessible for immunization. However, because the majority of commercial pigs are bred by artificial insemination (AI) (10), current husbandry practices allow routine access to the uterus during each reproductive cycle.

Adjuvant facilitate uptake of the antigen across the epithelial barrier, recruitment of APCs, activation of APCs, and they protect the antigen from degradation (1). One or several of these mechanisms of action may be required to generate a successful mucosal vaccine response and, therefore, the inclusion of multiple adjuvants may be necessary for an effective vaccine formulation (11). Certain mucosal surfaces have specialized epithelial cells such as M cells, which are efficient at sampling and delivering antigens to underlying immune cells and these cells can be targeted by adjuvants (4, 12). Although the uterine epithelia has no known specialized epithelial cells or canonically organized lymphoid tissue, it contains a multitude of epithelial cells and both luminal (13) and subepithelial lymphocytes (14). Thus, vaccine formulation and delivery need to be directed toward normal epithelial cells or at immune cells recruited to the uterine lumen or tissue.

The following study aims to determine which adjuvant components and combinations can generate an immune response in uterine epithelial cells (UECs). Additionally, we seek to determine if the inclusion of adjuvants in a semen dose modulates the uterine immune response to sperm and what role, if any, the UECs play in this response. Finally, we investigate whether delivering a vaccine during AI triggers an effective immune response in pigs. It is critical that any intrauterine vaccine administered during breeding does not have a negative effect on fertility or piglet growth kinetics.

## Materials and Methods

The majority of these methods are previously described in the thesis by Hamonic, University of Saskatchewan (15) and are presented here with permission.

### Animal Ethics

All experimental procedures were conducted in accordance with the guidelines of the Canadian Council on Animal Care (CCAC) under approval from the Animal Research Ethics Board at the University of Saskatchewan. Pigs were Landrace/Large White from Prairie Swine Centre, Inc. (PSC), a High Health herd that is free from porcine reproductive and respiratory syndrome virus, *Mycoplasma hyopneumoniae* and swine influenza virus. Pigs were housed in stalls for the duration of the experiments.

### Animal Trials and Sample Collection

#### Adjuvant Trial

Single parity sows were synchronized following a fixed-time AI protocol (16) prior to post-cervical insemination (Supplementary Figure 1). In brief, pigs were synchronized by oral progestin (Regu-mate; Merck Animal Health, USA) (17). Twenty-four hours after the final dose of oral progestin, pigs received 800 international units of pregnant mare serum gonadotrophin (Folligon; Merck Animal Health, USA) by intramuscular (i.m.) injection. Eighty hours later, pigs were given 5 mg porcine pituitary luteinizing hormone (Lutropin-V; Bioniche Animal Health, Belleville, ON) by i.m. injection (16). Thirty-two hours post-Lutropin-V injection, pigs were bred using post-cervical insemination catheters (Megapor) with a semen dose mixed with 3.2 ml of phosphate-buffered saline (PBS; Sigma Aldrich, Oakville, ON, Canada) (mock control sows, *n* = 3) or a standard semen dose containing 4 mg poly I:C (Invivogen, San Diego, CA, USA), 8 mg Host defense peptide 1002 (HDP, Genscript, Piscataway, NJ, USA), and 4 mg polyphosphazene (PCEP; Idaho National Laboratory, Idaho Falls, ID, USA) in 3.2 ml of PBS (TriAdj sows, *n* = 4). Adjuvants were administered into the opened semen bag then mixed by gentle inversion prior to being attached to the catheter for breeding. Sows were euthanized by captive bolt 24 h post-breeding and exsanguinated to allow necropsy of the reproductive tract and collection of uterine lavage. Small sections of tissue were collected from the cervix, lower uterine horn, mid uterine horn, upper uterine horn, ampulla, isthmus and ovaries for histology. Sections of the uterine horns were flash frozen in liquid nitrogen for RNA isolation and a duplicate section was frozen in Shandon cryomatrix (Thermofisher) for laser-capture microdissection collection.

#### Vaccine Trial 1

Sows used in this trial had previously received Porcine ParvoShield vaccine (Elanco Animal Health) by the i.m. route at each parity. The period between the last vaccination and the current intrauterine (i.u.) or i.m. immunization was at least 120 days. Sows were bred with semen alone or semen plus the vaccine (see below) using post-cervical catheters. Control sows (*n* = 3) received i.m. ParvoShield vaccine as they entered into farrowing crates (day 100 gestation) and they remained at PSC. Sows that were subjected to i.u. immunization (*n* = 4) were brought to VIDO-InterVac (Saskatoon, SK, Canada) prior to the start of the trial. The i.u. vaccine was comprised of 1 × 10^7^ TCID_50_ BEI-inactivated PPV (NADL-7; American Type Culture Collection) along with 400 μg poly I:C, 800 μg HDP, and 400 μg PCEP adjuvants (TriAdj) in 1 ml total volume, which were administered to the semen bag immediately prior to breeding. Sows were heat-checked twice daily after weaning by experienced personnel looking for a standard lordosis response following exposure to 5-α-androstenone (Hog-Mate; Reproduction Provisions, Inc., Walworth, WI, USA). Sows were inseminated with the AI dose alone or plus the vaccine 12 h after the first detection of lordosis (day 0) and then bred every 24 h with semen alone for the duration of the standing estrus. Blood was collected at day 0, 15, and 30 and then the i.u. vaccinated sows were humanly euthanized by captive bolt and exsanguination at day 30 post-vaccination. Reproductive tracts were externalized, the number of viable embryos in each uterine horn was recorded, and *corpus luteum* (CL) were counted as a measure of ovulation. Each fetus was visually inspected to establish whether they appeared viable to time of sow death.

#### Vaccine Trial 2

Gilts were administered oral progestin (Regu-Mate) for 14 days and then heat checked by experienced personnel using mature boars. Gilts were bred at the first sign of standing estrus by conventional AI with a standard semen dose with or without the vaccine and then every 12 h after with semen dose alone. The i.u. vaccine was comprised of 400 μg recombinant (r)VP2-Trx protein [cloned, expressed, and purified in *E. coli* as detailed in (6)] plus 400 μg poly I:C, 800 μg HDP, and 400 μg PCEP in 1 ml total volume (*n* = 7 gilts). Mock-vaccinated gilts (*n* = 9) received the standard semen dose and they were administered ParvoShield vaccine i.m. when they entered into farrowing crates at day 100 gestation. Blood serum was obtained at day 0, 15, 30, 70, 90, and at weaning. Piglet weights were obtained at day 3 and at day 21 from 6 randomly reselected gilts per group.

#### Vaccine Trial 3

Gilts were bred by cervical AI with a standard semen dose alone (control gilts, *n* = 5) or semen mixed with a combination of 3 separate vaccines (treatment gilts, *n* = 8). The i.u. vaccines were formulated with a consistent adjuvant dose of 266 μg poly I:C, 533 μg HDP and 266 μg PCEP combined with either 400 μg recombinant porcine epidemic diarrheal virus (PEDV) Spike protein, 200 μg recombinant *Lawsonia intracellularis* (LI) FliC protein or 1 × 10^7^ BEI-inactivated PPV. Recombinant FliC was purified from *E. coli* and rSpike protein was purified from HEK293 cells as detailed in Obradovic et al. (18) and Makadiya et al. (19), respectively. The control animals received i.m. injection with FarrowSure B Gold (Zoetis, Canada) to compare the anti-PPV vaccine response. Gilts were humanely euthanized after 30 days. The fetus viability relative CL numbers was presented as a ratio. The crown-rump ratio was measured using Image J and the average weight of the fetuses per litter was recorded.

### PBMC and Luminal Cell Processing

PBMCs were isolated from blood collected using EDTA Vacutainers (BD Biosciences) then centrifuged at 1,100 × g for 30 min. The buffy coats were collected and layered onto Ficol-Paque plus (GE life sciences) and centrifuged at 400 × g for 40 min. The PBMC layer was collected, washed in PBS 3 times with centrifugation at 250 × g for 10 min and stained for immunotyping by flow cytometry (described below) or stained with CFSE and restimulated with vaccine antigens (described below). The uterine horns were removed from the sows and flushed with 25 ml PBS + 1% BSA (Sigma-Aldrich) per horn to collect luminal cell populations, which were counted and stained for immunotyping by flow cytometry analysis and to quantify CCL2 (see below).

### Isolation, Culture, and Stimulation of Primary Uterine Epithelial Cells

Primary UECs were isolated from uterine tissue of gilts/sows collected from a local abattoir (*n* = 4) as described in detail in a previous study (20). Cells were polarized for 7–10 days as determined by stable 10x increase in transepithelial electrical resistance (TEER) with media changes taking place every second day. After cells achieved stable TEER, they were stimulated with 50 μg/ml poly I:C (Invivogen), 50 μg/ml lipopolysaccharide (LPS; *Salmonella enterica* serovar Minnesota from Sigma-Aldrich), 50 μg/ml CpG oligodeoxynucleotides (CpG 2395; Merial), 50 μg/ml muramyl dipeptide (MDP; Sigma-Aldrich), 100 μg/ml HDP (Genscript), 50 μg/ml PCEP (Idaho National Laboratory) or combined together in various combinations at the stated concentrations including as the triple combination adjuvant (TriAdj; poly I:C, HDP, PCEP). Six hours post-stimulation, cells were collected in Trizol (Invitrogen) for RNA extraction (described below).

### Sperm Abnormality and Mobility

Sperm abnormality assessment was performed on extended semen (PIC, Kipling, SK) alone or including the vaccine components from Trial 2 (individually or combined), which includes 1 × 10^7^ TCID_50_ binary ethylenimine (BEI)-inactivated PPV, 400 μg Poly I:C, 800 μg HDP 1002 and 400 μg PCEP. Extended semen alone or with the vaccine components was stored for 1, 3, 5, and 7 days at 17°C to mimic industry standard conditions. Alternatively, semen and components were warmed to 39°C with periodic readings for up to 360 min to assess how the extended semen alone or with the vaccine components were impacted at sow body temperature for a period of time after breeding. Sperm abnormality was assessed using multi-color flow cytometry to identify acrosome-reacted sperm by binding with peanut agglutinin (PNA) conjugated to Alexa-647 (Life Technologies). Sperm were stained with propidium iodide (BioVision, Milpitas, CA, USA) at a concentration of 5 mg/mL and PNA-Alexa647 at a concentration of 30 ng/mL, at room temperature for 5 min. Samples were then diluted 1:4 with Beltsville thawing solution (PIC) and 1 × 10^5^ events were collected using a FACSCalibur (BD Bioscience Franklin Lakes, NJ, USA) with analysis performed using FlowJo (Tree Star, Ashland, OR, USA). Dead sperm were identified if they were stained with propidium iodide. Experiments were repeated with three separate batches of semen.

Sperm motility was assessed for semen extended with Beltsville thawing solution alone or combined with 400 μg rPEDV spike protein, 200 μg rFliC protein, 1 × 10^7^ BEI-inactivated PPV and 800 μg poly I:C, 1,600 μg HDP and 800 μg PCEP (i.e., the cumulative components of Trial 4 vaccine). Sperm motility was evaluated following incubation for 30 min at 37°C and average motility across 5 unique fields of view were performed using an SCA CASA system for automatic sperm analysis.

### Porcine Parvovirus Propagation and Inactivation

PPV was propagated on fetal porcine testicular fibroblast testis (ST; CRL-1746) from American Type Culture Collection (Cedarlane, Burlington, Ontario, Canada). ST cells were cultured in Eagles minimal essential medium (Sigma) with the addition of 5% FBS (Gibco) and Antibiotic/Antimicotic (Life Technologies). Cells were hypotonically lysed in 0.01 M PBSA and free-thawed twice before removal of cell debris by centrifugation at 2,500 × g for 15 min. Viral particles were isolated from the resulting supernatant by centrifugation on top of a 25% sucrose cushion at 210,000 g for 2 h. Purification of the virus from the resulting pellet was carried out on a discontinuous gradient consisting of 1.2 and 1.4 M CsCl, centrifuged at 210,000 g for 1.5 h. Finally, the lower of the two resulting bands was collected and dialyzed against 3 changes of 10 mM Tris-HCl. The identity of the virus was confirmed by qPCR and TCID_50_ by serial infection of ST cells.

Inactivation of PPV was carried out with binary ethylenimine (BEI) following this published methodology (21). In short, BEI was prepared through the reaction of 0.1 M 2-bromo-ethylamine hydrobromide with 0.175 N NaOH at 37°C for 1 h with reaction validated colorimetrically with the addition of 0.0005% β-naphthol violet. Viral stock at 1 × 10^8^ TCID_50_/ml was inactivated with 1.5 mM BEI for 30 h at 37°C, before BEI was neutralized with 10 mM sodium thiosulfate. To confirm virus neutralization, inactivated PPV was passaged on ST cells for 5 passages with no evidence of CPE carried out both in house and by Prairie Diagnostic Services, Inc. (Saskatoon, Saskatchewan).

### Laser-Capture Microdissection Sample Collection

Cryoblocks were sectioned at 14 μm thickness onto polyethylene naphthalate membrane slides and immediately fixed in 70% ethanol. Residual cryomatrix was removed by submersion in DEPC treated water (Invitrogen), and slides were stained in cresyl violet (Sigma-Aldrich) for 30 s. Excess stain was removed by submersion in 70% and then 100% ethanol. Epithelial cells were captured within 45 min of staining using a PALM-Microbeam System (Zeiss), removing the basolateral third of the epithelial cell prior to capture to eliminate contamination of samples from sub-epithelial lymphocytes.

### RNA Isolation and Gene Expression Analysis

RNA analysis was carried out on both *uterine tissue (UTE) and laser captured uterine epithelia (LC-UE)* from gilts in Trial 1. Uterine tissue collected from the animal trial were ground at −80°C by mortar and pestle until the entire tissue section was reduced to a fine powder. Up to 100 mg of tissue was dissolved in 1 ml of Trizol (Invitrogen) for RNA extraction as detailed in Pasternak et al. (22). DNAse treatment was carried using the Turbo DNAse kit (Thermofisher) following the manufacturer's specifications and the inclusion of 10 units RNase inhibitor (Thermofisher). RNA quantity was determined by Nanodrop (Thermofisher) and RNA quality was validated by denaturing agarose gel. cDNA was generated from 2 μg of RNA using the high capacity cDNA kit (Thermofisher) following the manufacturer's specifications. Gene expression analysis was carried out on a StepOne Plus (Thermofisher) using KAPA SYBR mix (Sigma-Aldrich), containing 0.2 mM primer concentrations [primer sequences and annealing temperature used in Supplementary Table 1; (23–27)] and 10 ng/sample cDNA in 15 μl reactions run in duplicate.

For gene expression analysis from laser-captured uterine epithelial cells (LC-UE), RNA was isolated using the Picopure RNA isolation kit (Thermofisher) following the manufacturer's specifications including an on-column DNase treatment (Qiagen). RNA quantity and integrity were confirmed using the Bioanalyzer (Agilent) and 200 ng RNA per sample was converted to cDNA using the High-Capacity cDNA Reverse transcription kit as described above. Gene expression analysis was carried out as described above using 4 ng/sample in each reaction. Primer amplification efficiency was measured at the optimal annealing temperature and in all instances was found to be >90%. Gene expression was normalized to the geometric mean of multiple stable reference genes, RPL19, YWHAZ and GAPDH for the *in vitro* analysis, and GAPDH and β-Actin for *in vivo* analysis (Supplementary Table 1).

### Immunotyping of PBMCs and Cells Obtained by Uterine Flush

Cells collected from uterine flush were washed 2x in PBS + 0.1% EDTA at 400 × g for 15 min and counted by a coulter counter (Beckman Coulter). Both PBMCs and cells flushed from the uterine tissues (from Trial 1) were stained for flow cytometry (FCM) analysis in 96 well plates with 1 × 10^6^ cells/wells. All FCM stains were incubated in stains diluted in PBS + 2% FBS for 10 min at room temperature followed by 3x washes in PBS + 2% FBS centrifuging at 500 × g for 3 min. All antibody concentrations and details are available in Supplementary Table 2. PBMC and flushed T cells were stained in a four-step staining procedure beginning with anti-CD4, anti-CD8α and anti-TCRγδ, followed by the secondary antibodies anti-IgG2b-FITC, anti-IgG2a-Alexa 647, and anti-IgG1-biotin. Next, IgG and Streptavidin (SA)-PerCP-Cy5.5 was added, followed by the directly labeled anti-CD3-PE antibody. PBMCs and flushed B cells were stained with anti-CD21 followed by anti-IgG1-APC. PBMC monocytes were stained with anti-CD172 and anti-CD14, followed by anti-IgG1-PE and anti-IgG2b-APC. Flushed myeloid cells were stained with anti-CD172, anti-MHCII, anti-SWC9, and anti-CD16, followed by anti-IgG2b-FITC, anti-IgG2a-PE, and anti-SA-PerCP-Cy5.5. FCM samples had 60,000 events for PBMCs and 250,000 events for flushed cells, all of which were immediately collected on a FacsCalibur (BD) with appropriate fluorescence minus one (FMO), single stains, and isotype stains. FCM analysis was carried out using FlowJo (FlowJo LLC). A representative flow cytometry gating scheme for blood analysis (and luminal cell lymphocytes only) is shown in Supplementary Figure 2, such that CD3^−^CD8α^+^ represent natural killer (NK) cells, CD3^+^TCRγδ^−^CD4^+^CD8α^−^ represent CD4^+^ T cells, CD3^+^TCRγδ^−^CD4^−^CD8α^+^ represent CD8^+^ T cells, CD3^+^TCRγδ^−^CD4^+^CD8α^+^ represent CD4^+^CD8^+^ T cells, and CD21^+^ represent B cells. A representative gating scheme for the flushed myeloid cells is shown in Supplementary Figure 3, such that CD172^+^MHCII^−^CD16^+^ cells represent neutrophils, and CD172^+^MHCII^+^SWC9^−^ cells represent APCs.

### CCL2 ELISA

Uterine horn luminal CCL2 was quantified by sandwich ELISA against porcine CCL2 (Kingfisher Biotech) following manufacturer's instructions. In short, 96 well high binding plates (Immulon II, VWR) were coated with a polyclonal anti-swine CCL2 (Kingfisher Biotech) at 1 μg/ml in PBS overnight at RT. Plates were then blocked by 4% BSA in PBS for 2 h at RT prior to a 1 h RT incubation with CCL2 standard (1 in 2 dilutions from 10 ng/ml to 10 pg/ml) and undiluted flush samples. Plates were washed with TBST and biotinylated anti-swine CCL2 antibody was incubated at 0.5 μg/ml in PBS + 4% BSA for 1 h at RT followed by washing and a 30 min RT incubation with streptavidin-HRP. Plates were developed with TBS for ~30 min in the dark before stopping with a 2N sulfuric acid and absorbance was read at 450 nm.

### Antibody ELISAs

Antibody ELISAs were performed on serum and on supernatants from uterine tissue finely minced then incubated in AIM-V media for 48 and 120 h. To measure antibody response to BEI-inactivated PPV, rVP2-TRx, and/or rFliC, Immulon II plates (VWR) were coated over night at with 0.6 μg/ml rVP2-TRx (6) or 2 μg/ml rFliC protein in coating buffer. Plates were washed with tris-buffered saline with 2% Tween-20 (TBST). When detecting antibodies against rPEDV protein, Immulon plates were coated with 0.5 μg/ml purified rSpike S1 protein in coating buffer. Plates were washed with TBST + 0.1% Tween 20.

For all ELISAs, sera and supernatants from minced tissues were serially diluted in assay diluent buffer TBST (+ 1% fish gelatin for the rSpike S1 protein ELISA only). After 2 h incubation, the plates were washed in TBST then incubated for 1 h with 1/5,000 Alkaline phosphatase-conjugated Goat anti-Pig IgG (H+L) (KPL catalog #151-14-06). ELISAs were then developed with 1 mg/ml p-nitrophenyl phosphate in DE buffer (1 M diethanolamine, 0.5 M magnesium chloride) and absorbance at λ405 nm was measured on a SpectraMax plus microplate reader (Molecular Devices). All end-point titers were determined using 4-fold serial dilutions with initial dilutions of serum and culture supernatants performed at 1:4.

### Histology and Immunohistofluoresence

Small sections of tissue were collected from the gilts (Trial 1) cervix, lower uterine horn, mid uterine horn, upper uterine horn, ampulla, isthmus, and ovaries and fixed in formalin for 36 h. Formalin-fixed tissue was processed and embedded into paraffin blocks that were sectioned at 4 μm and floated onto superfrost plus slide (Thermofisher). Tissue blocks were deparaffinized by xylene and rehydrated by decreasing concentrations of ethanol prior to Haemotoxylin and Eosin (H&E) staining.

Duplicate slides were deparaffinized and rehydrated from the middle uterine tissue for anti-CD163 immunohistofluoresence (IHF) wherein the slides underwent heat-mediated antigen retrieval in 10 mM Na-Citrate, pH 6 for 30 min at 90°C before being blocked in 5% skim milk in TBS for 1 h at room temperature. Primary antibody staining with mouse anti-human CD163 (EdHu-1; Bio-Rad) at 10 μg/ml in dilution buffer (PBS with 1% BSA, 1% horse serum, 0.3% triton-X, and 0.01% sodium azide) overnight at 4°C. Slides were washed 3x in TBS + 0.05% Tween 20 and incubated in 5 μg/ml donkey anti-mouse IgG Al555 (Invitrogen) for 90 min at room temperature. Slides were again washed 3x in TBS + 0.05% Tween 20 and then stained with 4′,6-diamidino-2-phenylindole (DAPI; Invitrogen) in methanol for 10 min before being cover slipped and imaged on Axiovert 200M (Zeiss) at 20x magnification with appropriate isotype controls. CD163 positive cells were counted in ImageJ by analyze particles, selecting particles between 100 and 1,000 pixels and identified cells were confirmed manually.

### Statistical Analysis

All statistical analysis was carried out using GraphPad Prism 7 (GraphPad Software). Gene expression analysis of *in vitro* UEC stimulations were evaluated by one-way ANOVA and significant differences between mock-treated cells and individual treatments were determined by Holm-Sidak's multiple comparisons test. Gene expression and blood immunotyping from *in vivo* experiments and weights of newborn and weaners, fetus to CL ratios, average length of crown/rump ratio per litter were evaluated by unpaired *t*-test with Welch's correction. Uterine flush immunotyping was evaluated by Mann Whitney test. CD163 recruitment analysis was evaluated by unpaired *t*-test with Welch's correction. In all cases, significant differences were reported by ^*^p < 0.05, ^**^P < 0.01, and ^***^P < 0.001.

## Results

### Cytokine and Chemokine Gene Expression Changes in Uterine Epithelial Cell in Response to Stimulation With Adjuvants

We first evaluated the potential impact of vaccine adjuvants on the uterus through *in vitro* culture with primary epithelial cells. Following stimulation of UECs with adjuvants alone or in combination, the cells stimulated with poly I:C-HDP and poly I:C-HDP-PCEP showed TEER values that dropped significantly at 6 h (Supplementary Figure 4A). TEER values returned to initial levels by 24 h post-stimulation (Supplementary Figure 4B) which suggest that these combinations of adjuvants may transiently impact tight-junction integrity.

**Figure 1:**
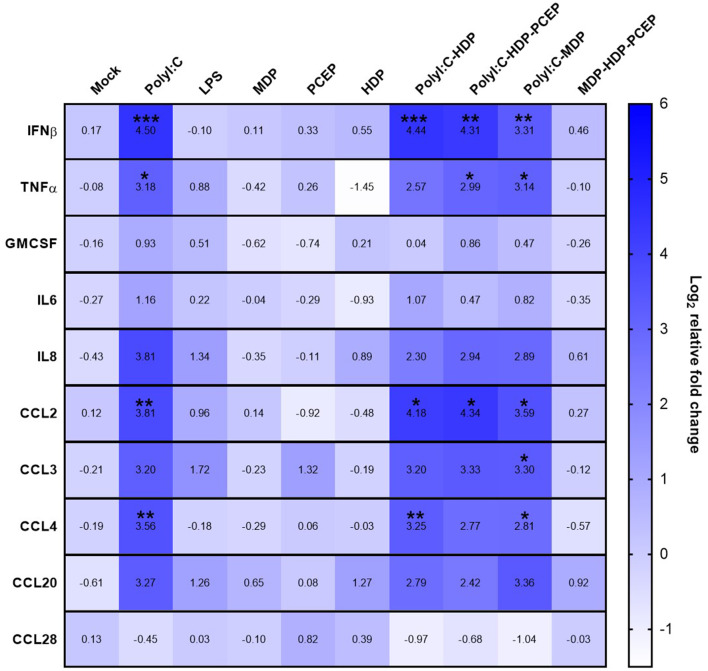
Gene expression heat map of polarized uterine epithelial cells (UECs) stimulated with multiple adjuvant components alone and in combination. UECs were cultured until polarized and stimulated by adjuvant components (horizontal axis) for 6 h before cells were collected, RNA was isolated and gene expression was analyzed by qPCR. Median log_2_ increases are presented in the heat map with significant differences were evaluated by one-way ANOVA and significant differences between mock-treated cells and individual treatments were determined by Holm-Sidak's multiple comparisons tests (*p < 0.05, **p < 0.01, and ***p < 0.001).

Compared to mock-stimulated UECs, poly I:C significantly increased UEC expression of IFNβ (4.5-fold increase, *p* < 0.0005), TNFα (3.18-fold increase, *p* < 0.03), CCL2 (3.81-fold increase, *p* < 0.005), and CCL4 (3.56-fold increase, *p* < 0.007) but poly I:C did not significantly increase expression of GM-CSF, IL-6, IL-8, CCL3, CCL20, or CCL28 (Figure 1). Stimulation of UECs with LPS, MDP, PCEP, HDP alone, or MDP-HDP-PCEP in combination did not significantly impact the expression of any of the evaluated immune response genes. When poly I:C was co-incubated with other adjuvants, there was a significant change in gene expression relative to the mock-stimulated cells, but no differences relative to poly I:C alone. For example, poly I:C-HDP stimulated UECs showed significantly induced IFNβ (4.44-fold increase, *p* < 0.0004), CCL2 (4.18-fold increase, *p* < 0.01), and CCL4 (3.25-fold increase, *p* < 0.006) gene expression relative to the mock-stimulated cells. The poly I:C-HDP-PCEP and poly I:C-MDP stimulated UECs showed significantly induced expression of IFNβ gene (4.31, *p* < 0.003 and 3.31-fold increase respectively, *p* < 0.002), TNFα (2.99, *p* < 0.04 and 3.14-fold increase, *p* < 0.04, respectively), and CCL2 (4.34, *p* < 0.02 and 3.3-fold, *p* < 0.02 increase, respectively). Stimulation of UECs with poly I:C-MDP in combination significantly induced CCL4 (2.81-fold increase, *p* < 0.04) and was the only treatment able to significantly induce CCL3 expression (3.3-fold increase, *p* < 0.05) relative to the mock-stimulated UECs, although non-significant, equivalent numerical changes were noted in all other treatments which included poly I:C. No adjuvants significantly induced the expression of GM-CSF, IL6, and CCL28 when compared to the mock stimulation. SLA-DRA gene expression was not detected in any UEC stimulation sample (data not shown) indicating porcine UECs do not express MHC class II.

### Impact of Semen and Adjuvants on Uterine Luminal Cell Populations and PBMC Composition After Breeding

**Figure 2:**
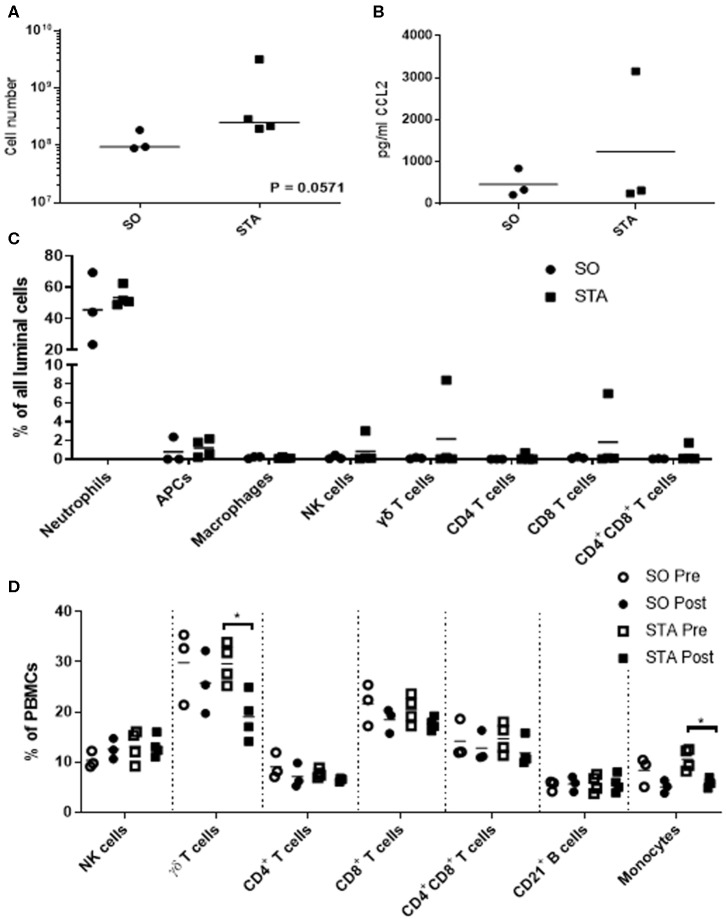
Uterine flush cell counts and immunotyping of luminal cell populations of sows 24 h after breeding with semen only (SO) or semen containing a triple adjuvant combination (STA) in addition to PBMC immunotyping before or 24 h after breeding. Flushed cells were counted by coulter counter **(A)**. Luminal CCL2 was quantified by sandwich ELISA **(B)** and significant differences between treatments were determined by Mann Whitney test. Immunotyped cells in the uterine flush were stained with CD3, CD4, CD8α, γδ T cells, CD172, MHCII, SWC9, and CD16 **(C)**. PBMCs were isolated from blood and stained for CD3, CD4, CD8α, γδ T cells, CD21, CD172, and CD14 **(D)**. Stained cells were analyzed on a FACScalibur and significant differences between treatments determined by Mann Whitney test. Each circle or square represents a unique biological replicate and the line represents mean data. *p > 0.05.

Because we are interested in understanding how adjuvants administered with semen impacts the pig uterus, our next steps were to measure changes in luminal cell population 24 h post-breeding with semen alone or semen plus adjuvants. We selected three adjuvants (4 mg poly I:C, 4 mg PCEP, and 8 mg HDP; TriAdj) to use in combination. Sows were administered semen +/− TriAdj and we observed that the semen spiked with TriAdj (STA) triggered a non-significant trend in increased luminal cells (*p* = 0.057) compared to the number of luminal cells in sows administered semen only (SO) (Figure 2A). To determine whether the changes in CCL2 gene expression analysis observed in polarized UECs stimulated with TriAdj (Figure 1) correlates to increased CCL2 secretion 24 h after breeding with STA relative to SO, we quantified CCL2 secretion from uterine flushes and saw no significant differences (Figure 2B). STA did not significantly impact CCL2 luminal secretion by luminal cells which could indicate a lack of protein translation or that secretion of CCL2 was directed into the tissue as opposed to into the lumen.

To determine whether inclusion of TriAdj with the semen dose impacted cell recruitment to the uterus, we enumerated total cells collected from the uterine lumen 24 h after breeding with SO or STA. The most predominant cell populations in the uterine lumen following breeding were neutrophils with mean population percentages at 45% total events in response to SO and 53% of total events in response to STA, followed by non-macrophage APCs at 0.79% total events in response to SO and 1.19% total events in response to STA, respectively (Figure 2C). All other cell populations were below 1% of total events, regardless of treatment with the exception of one animal bred with STA which had higher total events for NK (3%), γδ T cells (8.4%), and CD8 T cells (6.98%). Overall, the inclusion of TriAdj in semen did not appear to significantly impact the proportions of immune cell populations in the uterine, although there was a trending increase in the total number of cells collected (*p* = 0.0571).

We performed immunotyping on PBMCs to discern whether the number of T cell subsets, B cells, and monocytes were impacted by either breeding (i.e., pre-semen vs. post-semen; pre-semen + TriAdj vs. post-semen + TriAdj) or by the adjuvants administered to the uterus during breeding (SO vs. STA). Before and after breeding with SO or STA, there was no significant change in the percentages of the blood cell population of CD3^−^CD8^+^ NK cells, CD4 T cells, CD8 T cells, CD4^+^CD8^+^ co-positive T cells, or CD21^+^ B cells (Figure 2D). After animals were bred with STA, there was a significant drop in the percentage of γδ T cells (10.5% decrease) and monocytes (4.7% decrease) in the PBMC mixed cell populations relative to the percentages present in PBMCs prior to STA immunization suggesting that the TriAdj may have impacted blood cell composition. However, when we compared the blood cell populations in sows bred with semen vs. sows bred with semen plus TriAdj, we did not observe significant differences in any of the population percentages.

### CD163 Positive Cell Recruitment to Uterine Tissue Following Breeding

To determine if the decreased monocytes in blood in response to STA (shown in Figure 2D) shows a corresponding influx of CD163 positive monocytes into uterine tissue, immunohistofluorescence was carried out on sections from the middle of the uterine horn (representative staining in Supplementary Figure 5A). CD163 positive cells were enumerated per 100 μm^2^ section. No significant differences in the number of CD163^+^ cells were found in the uterine tissue from sows bred with SO (1.23 cells per 100 μm^2^) or sows bred with STA (2.03 cells per 100 μm^2^; Supplementary Figure 5B).

### Impact of Semen Alone or Semen Plus Adjuvants on Uterine Tissue and Laser-Captured Uterine Epithelial Cell Gene Expression

Twenty-four hours after sows were bred with semen alone or semen plus Triadj, the uterine tissue (UT) from lower to upper uterine horns were subjected to gene expression analysis. Relative to the UT exposed to SO, UT exposed to STA did not result in significant differences in expression of TNFα, IFNβ, GM-CSF, IL-6, IL-8, CCL2, CCL3, CCL4, or CCL28 genes (Supplementary Figure 6).

We speculated that we may not be able to discern whether gene expression profiles of the uterine epithelial cells were being masked by the expression profiles of the multiple cell populations present in UT. Therefore, we performed laser-capture microdissection (LCM) such that we captured only the uterine epithelial cells (LC-UEs). LCM was performed on cryoblocks from only the middle of the uterine horn as no significant differences in gene expression were observed between lower, middle and upper uterine horn UT. LC-UE cells from animals bred with SO or STA also showed no changes in expression of TNFα, IFNβ, GM-CSF, IL-6, IL-8, CCL3, CCL4, or CCL28 (Supplementary Figure 6). However, the LC-UE cells isolated from sows bred with STA showed significantly induced expression of CCL2 (2.4-fold increase; *p* < 0.0274) relative to the expression profile observed in LC-UE cells from sows bred with SO. Lastly, SLA-DRA gene expression was not detected in the LC-UE samples and had no significant differences when observed in tissue (data not shown). Collectively, these data suggest that TriAdj administered with semen during breeding had an impact on select uterine epithelial cell chemokine expression. Our next steps were to determine whether i.u. vaccination with the TriAdj triggered an immune response.

### Response to Intrauterine Vaccine Administered With Semen at the Time of Breeding

**Figure 3:**
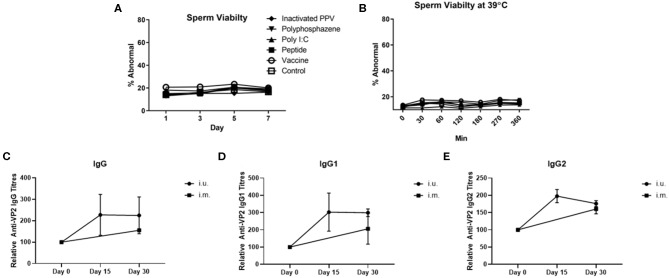
Sperm abnormality assessment of extended semen incubated with intrauterine (i.u.) vaccine components and fetus viability after i.u. immunization and serum antibody titers from animals vaccinated through the i.u. or intramuscular routes (i.m.). Commercially extended semen was incubated alone or in the presence of 1 × 10^7^ TCID_50_ BEI-inactivated PPV, 400 μg Poly I:C, 800 μg HDP, 400 μg PCEP (i.u. vaccine) at 17°C for 7 days **(A)** or 39°C **(B)** with periodic readings for up to 360 min. Acrosome-reacted sperm was bound by peanut agglutinin (PNA) conjugated to Alexa-647 and identified on a FACScalibur. Dead sperm were identified if they were stained with propidium iodide (PI). Experiments were repeated with three separate batches of extended semen. **(C–E)** Animals were bred with extended semen alone or with i.u. vaccine. Control sows (*n* = 3) were immunized with ParvoShield vaccine by i.m. route. All sows had previously been vaccinated i.m. with ParvoShield at each breeding cycle when they entered into the farrowing crates (~120 days previously). Serum anti-VP2 IgG **(C)**, IgG1 **(D)** and IgG2 **(E)** antibody titres over time were quantified relative to each sow's anti-VP2 titres at day 0 to give relative anti-VP2 titres for i.u.-vaccinated (black circle) and i.m.-vaccinated (black square) sows. Data are presented as means [horizontal bars; **(A,B)**] and mean with standard deviation in **(C–E)**.

For our first animal trial, the i.u. vaccine was comprised of 1 × 10^7^ TCID_50_ BEI-inactivated PPV vaccine formulated with 400 μg Poly I:C, 800 μg HDP and 400 μg PCEP. Prior to vaccination we evaluated the impact of this formulation on sperm and found no significant effect on either acrosome reaction or viability during storage for 7 days (Figure 3A) or at physiological temperatures over 360 min incubation (Figure 3B). Flow cytometric analysis showed that the vaccine components alone or in combination had no significant impact on the percentage of abnormal semen. Next, treatment sows (*n* = 4) were bred with semen combined with the vaccine immediately prior to breeding. Control sows (*n* = 3) were immunized with ParvoShield vaccine by i.m. route when they entered into farrowing crates. All sows had previously been vaccinated i.m. with ParvoShield at each breeding cycle when they entered into the farrowing crates (~120 days previously) so we are measuring a booster vaccine response. Serum was tested for anti-VP2 antibodies up to 30 days later. Results showed that sows responded to the i.u. vaccine with anti-VP2 IgG (Figure 3C), IgG1 (Figure 3D), and IgG2 (Figure 3E) titres that were comparable to the titres from sows immunized with the commercial i.m. PPV vaccine. The individual antibody titres for each animal is shown in Supplementary Figures 7A–C and the data shown as percentage change from the zero time point is shown in Supplementary Figures 7D–F. Together these results show that the i.u. vaccine did not negatively affect sperm function or embryo viability and that sows responded to an inactivated PPV vaccine administered with the semen dose with elevated serum anti-VP2 titres if the sows had previously received an i.m. porcine parvovirus vaccine.

For our second trial, we immunized gilts via the i.u. route (*n* = 7) with 800 μg rVP2 antigen with 400 μg Poly I:C, 800 μg HDP and 400 μg PCEP. Mock-control sows (*n* = 9) were administered a comparable volume of saline with the semen dose. Serum was obtained throughout gestation and continued until weaning (21 days after birth). Piglets born from i.u. vaccinated gilts (*n* = 6 randomly selected) had comparable weights at 3 days of age (Supplementary Figure 8A) and at weaning (Supplementary Figure 8B) relative to the piglets born from mock-vaccinated dams (*n* = 6 randomly selected) suggesting that the i.u. vaccine components did not negatively affect piglet development. Serum anti-VP2 IgG titres were at comparable low levels across all time points with no significant differences between the 2 groups (Supplementary Figure 8C) suggesting that either rVP2 was a poor antigen or that the i.u. vaccine was not effective as a primary vaccine.

**Figure 4:**
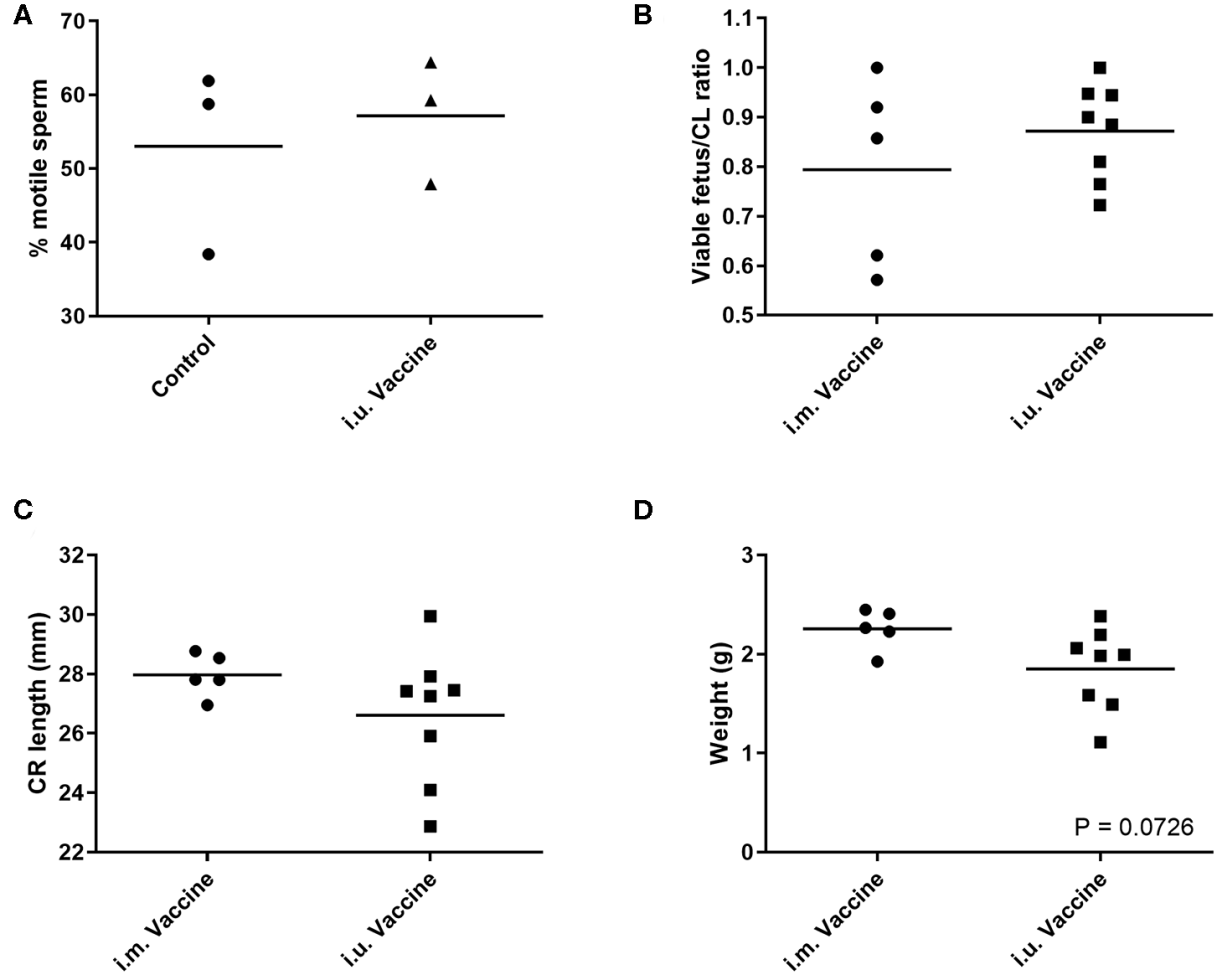
Sperm motility measurements of extended semen incubated with vaccine and fetal morphometrics from animals vaccinated through the intrauterine or intramuscular routes. **(A)** Sperm motility was evaluated in the presence of inactivated PPV, Spike and LI FliC (i.u. vaccine) and TriAdj using SCA CASA system for automatic sperm analysis and the average motility across 5 unique fields of view. **(B–D)** Fertilization rates and fetal morphometrics were measured 30 days after breeding following i.m. vaccination with Farrowsure B Gold vaccine (which contains PPV antigens) or i.u. vaccination with 3 vaccines each consisting of 400 μg recombinant PEDV spike protein, 200 μg recombinant LI FliC protein, and 1 × 10^7^ BEI-inactivated PPV each formulated with 266 μg poly I:C, 533 μg HDP and 266 μg PCEP. **(B)** The ratio of viable fetuses divided by the CL per sow are presented. **(C)** The distance in mm between the crown and rump was measured for each fetus and the ratio are presented. Each data point represents the average length for the fetuses born to each gilt. **(D)** The average weight of the fetuses (g) from each litter are presented. Statistical analysis carried out by Kruskal-Wallis test and Dunns multiple comparisons test. Horizontal bars represent mean values.

**Figure 5:**
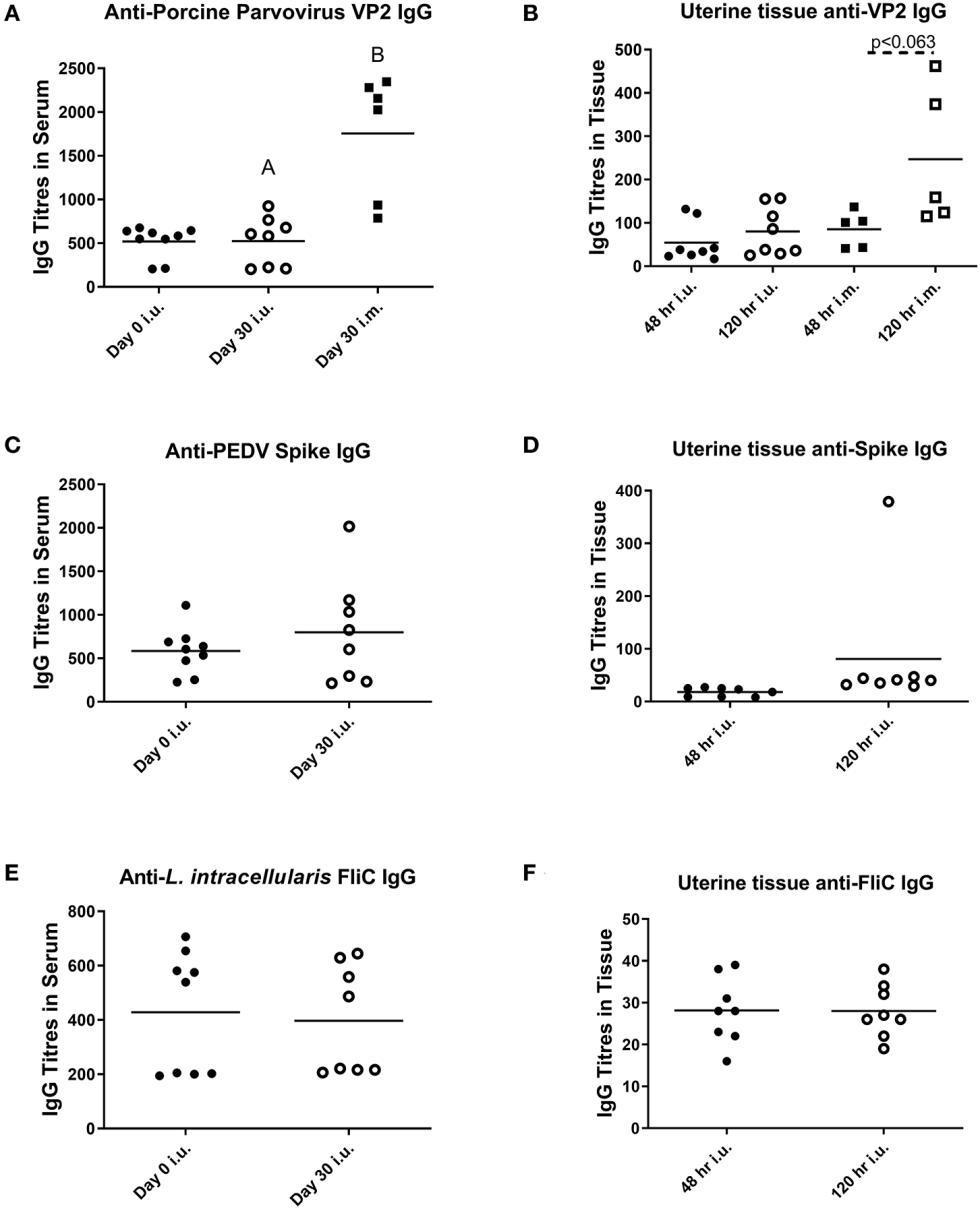
Serum and mucosal antibody titers from animals vaccinated through the intrauterine or intramuscular routes. Serum **(A,C,E)** and mucosal antibody titers **(B,D,F)** were measured after breeding animals with semen alone then immunizing them through the i.m. route with Farrowsure B Gold vaccine (which contains PPV antigens) or after breeding animals with semen combined with 3 vaccines consisting of 400 μg recombinant PEDV spike protein, 200 μg recombinant LI FliC protein, and 1 × 10^7^ BEI-inactivated PPV each formulated with 266 μg poly I:C, 533 μg HDP and 266 μg PCEP. Serum was collected at day 0 and 30 days later and uterine tissue was collected at day 30 after gilts were humanely euthanized. The supernatants from the minced uterine tissues was collected after 48 and 120 h to establish mucosal antibody production. Data are presented as mean values. Statistical analysis carried out by Kruskal-Wallis test and Dunns multiple comparisons test. Significantly different groups are denoted by differing letters.

For our third trial, we combined semen with TriAdj and one of three antigens including rPEDV Spike protein, rFliC, and BEI-inactivated PPV. We performed CASA analysis to assess sperm motility and we observed no difference in the percent motile sperm between semen alone or semen incubated with the vaccines (Figure 4A). The two vaccine groups consisted of i.u.-vaccinated sows (*n* = 8) and control sows (*n* = 5) which were immunized with parvovirus vaccine FarrowSure B Gold i.m. at breeding. After 30 days, fetuses were visually inspected and the CL were counted. There was no difference in the viable fetus/CL ratio between both groups of sows (Figure 4B). The length of the fetus from the crown to the rump (mm) was measured for each fetus and the average crown-rump length was comparable across both groups of sows (Figure 4C). There was no significant difference in the average fetus weight born to either groups of sows (Figure 4D). Collectively, these data indicate that the vaccines comprised of recombinant proteins or inactivated PPV vaccine each formulated with TriAdj did not negatively affect sperm function or fetus viability, fetal crown-rump length, or birth weight in the i.u. vaccinated sows relative to the control sows. Finally, we assessed the impact of the anti-VP2 response in the sow sera and uterine tissue immune responses (Figure 5). Thirty days post-immunization, serum anti-VP2 IgG were assessed and we observed that the animal immunized with Farrowsure B Gold vaccine i.m. had a significant increase in antibody titres relative to the i.u. vaccinated gilts after 30 days (Figure 5A). Similarly, when the uterine tissues were minced and incubated in media for 48 and 120 h to allow measurement of local antibody production, only the i.m. vaccinated animals showed a statistically not-significant (*P* < 0.063) increase in anti-VP2 IgG titres (Figure 5B). The serum and mucosal antibody titres for i.u. vaccinated gilts were also calculated for the other two antigens included in the i.u. vaccine, rPEDV Spike and rFliC protein (which are absent in Farrowsure B Gold vaccine). There was no significant increase in anti-PEDV Spike IgG in serum (Figure 5C) or uterine tissue (Figure 5D) or anti-FliC IgG in serum (Figure 5E) or uterine tissue (Figure 5F). These data suggest that a primary vaccine comprised of BEI-inactivated PPV or recombinant proteins formulated with TriAdj administered to the uterus at breeding failed to promote a systemic or mucosal humoral immune response.

## Discussion

Initiating a strong mucosal immune response to inactivated virus or subunit vaccines requires potent adjuvants that overcome the mucosal barriers and initiate recruitment of APCs to the mucosal surface. As the uterine epithelial layer is the first cellular contact for an i.u. vaccine, generating a strong chemoattractive response that leads to APC recruitment to the uterine tissue or the uterine lumen may increase i.u. vaccine efficacy. Immunostimulatory adjuvants frequently considered for use in mucosal vaccines are TLR agonists and other pattern recognition receptor ligands that act through the inflammasome. Although porcine UECs express the necessary receptors for all the ligands evaluated [TLR3 bound by poly I:C, TLR4 bound by LPS, TLR9 bound by CpG, NOD2 bound by MDP (28)], our study showed that these cells only induced expression of the pro-inflammatory cytokine IFNβ and TNFα and chemokine genes CCL2 and CCL4 in response to poly I:C suggesting that TLR3 was a viable adjuvant target. This analysis shows agreement with our previous research which also showed that pig uterine epithelial cells express functional TLR3 which is targeted by poly I:C (20). *In vitro* experiments have shown poly I:C and LPS stimulation of murine UECs significantly induced secretion of CCL2, while CpG stimulation was unable to induce CCL2 expression (29). In contrast, LPS stimulated Human UECs showed suppressed CCL2 expression whereas poly I:C induced secretion of TNF-α, GM-CSF, IL-6, G-CSF, CCL2, and CCL4 (30, 31). Lastly, although porcine UECs showed induced expression of pro-inflammatory cytokine and chemokine genes in response to poly I:C, LPS stimulation had no observable impact on the assayed genes. These results suggest that poly I:C alone or in combination may be a suitable adjuvant to use to target uterine epithelial cells innate immune responses. The notable discrepancies of responses between species supports the concept that although TLR expression in UECs is relatively conserved across species, the response upon TLR ligand stimulation between species can vary significantly and caution should be taken in attempting to extrapolate results across species.

Non-TLR ligands are less regularly evaluated as adjuvants, however, porcine UECs express the receptors for several potential adjuvants such as NOD2, the receptor for MDP which may indicate that NOD2 may be a suitable adjuvant (28). Although there are no studies showing significant *in vitro* stimulation of UECs with MDP, *in vitro* studies with mouse APCs showed minimal NF-κβ activation unless MDP was combined with other ligands such as CpG (32). Our results show that pig UECs did not induce expression of any assayed genes in response to MDP alone nor did MDP amplify the response generated toward poly I:C. Therefore, we do not anticipate that it will be an effective adjuvant in inducing APC recruitment or activation in an i.u. vaccine. HDP, which has no known receptor, has been implicated in modulating the immune response in several cell types including monocytes where *in vitro* stimulation resulted in increased CCR5 expression and enhanced recruitment to CCL3 and CCL5 (33). Although there has been observed HDP modulated activity in other cells, both when alone and combined with other adjuvant components, HDP showed no significant impact on the capacity for porcine UECs to respond to poly I:C. Lastly, there have been studies evaluating polyphosphazene in both mucosal and parenteral vaccine formulations where PCEP alone induced protective immune responses (34). Intramuscular injection of mice with PCEP triggered local production of CCL2 and pro-inflammatory cytokines as IL-1beta, and IL-18 cytokines and when injected intradermally into pigs, PCEP induced the expression of chemokine CCL2 and pro-inflammatory cytokine IL-6 suggesting that it has immunostimulatory potential (35, 36). These observations suggest that PCEP can act as an immunostimulatory adjuvant and it may potentiate immune responses to antigens. Despite these results in mice and pigs after parenteral injection/vaccination, porcine UECs stimulated with PCEP did not induce expression of cytokine or chemokine genes and may not be an effective i.u. vaccine adjuvant alone.

TriAdj as a vaccine adjuvant has been evaluated in multiple vaccine formulations, in multiple species, and delivered via several routes. Primarily it has been evaluated for use as an i.m. vaccine adjuvant where it has been used in mice, rats, cattle, sheep, and pigs generating strong systemic immunity against human parainfluenza type 3 (in mice and rats), bovine viral diarrhea virus (in cattle and sheep) and porcine epidemic diarrhea virus (in pigs) (11, 19, 37). TriAdj has also been used to generate a strong single dose humoral and cell-mediated immune response when delivered subcutaneously in koalas as a subunit chlamydia vaccine (38). When TriAdj was used in conjunction with mucosal vaccine studies, there was increased mucosal immunity and protection generated to an intranasal vaccine to respiratory syncytial virus in mice (39). A promising use for the TriAdj as a mucosal adjuvant was shown when it was administered as part of a subunit vaccine in the rabbit uterus as it induced strong systemic and mucosal humoral immune responses even after a single dose (6). Although there have been limited studies on the initial innate immune response generated to TriAdj, an *in vitro* study with mouse macrophages found that they induced significant expression of several chemokines including CCL2, CCL3, and CCL4 in addition to upregulation of the co-stimulatory molecules CD80/86 and MHC class II (40) in the presence of TriAdj.

Because i.u. vaccination in commercial sows would only be used during AI, it is important to take into account the immune response generated during breeding. Breeding in swine elicits an inflammatory immune response and neutrophil infiltration into the uterine lumen (41, 42). However, with the exception of a widely accepted IL-8 induction and corresponding polymorphonuclear cell recruitment to the lumen (43, 44), there are limited studies examining the exact cytokine and chemokine genes induced following breeding. Interestingly one previous study showed that the semen extender Androhep and seminal plasma alone induced IL-10, TGF-β, IL-8, and TNF-α, however when combined with spermatozoa, these values returned to baseline expression levels (45). The possible suppression of cytokine and chemokine expression by spermatozoa may contribute to the discrepancy in the magnitude of expression observed *in vivo* that was lower than what was observed in the *in vitro* experiments. However, studies evaluating immune cell recruitment into the endometrium following breeding remain somewhat unclear whether spermatozoa, seminal plasma, or semen extender is the primary inducer of this response (43). We speculate that this inflammatory response may reduce the requirement of an i.u. vaccine to induce an inflammatory response itself, and may instead require the adjuvants to modulate the inflammatory response toward a higher proportion of recruited APCs in the uterine mucosa, possibly through the induction of chemokines that will preferentially recruit APCs, such as CCL2 and CCL3. In pigs bred with semen alone or semen plus TriAdj, we observed increased expression of CCL2 and CCL4 genes but no detectable increase in luminal CCL2 protein. While it is possible that CCL2 is secreted by the uterine epithelia basolaterally, we would anticipate observing a greater degree of APC recruitment into the endometrium if this were the case. Further, despite the increased expression of chemokines known to promote monocyte and macrophage recruitment chemokines as well as decreased levels of monocytes in the blood, we did not observe a significant increase in the numbers of monocytes/macrophages (CD163 positive cells) in the uterine tissue when compared to the response to extended semen. Although there are numerous studies characterizing the polymorphonuclear cell recruitment into the lumen following breeding and the inflammatory response following breeding with extended semen (41, 42, 45), data on APC recruitment in swine is limited. However, a single study observed increased MHCII expression on uterine macrophages and DCs following breeding, indicative of APC maturation (44). These data and the non-significant decrease of blood monocytes after breeding in our study may be indicative of a certain degree of APC engagement to extended semen alone and inclusion of TriAdj in semen although more research is required to understand this.

Previous studies have described that the lumen of the porcine uterus, in a native state, has a relatively low-level complement of T cells (13) which is consistent with our observations. Further, our data shows that not only does semen plus TriAdj not impact T cell recruitment to the uterine lumen, we also show that breeding appeared to have minimal effect on luminal T cell numbers. It remains to be clarified why blood γδ T cells were reduced after animals bred with semen plus TriAdj but not in animals bred with semen alone and why there is no evidence that the γδ T cells were recruited to the uterine lumen. Current data indicate that circulating porcine γδ T cells are primarily pro-inflammatory (46) and therefore further research should be carried out to determine if the inflammatory response induced by TriAdj plus semen is specifically recruiting these cells. Based on the limited data available for γδ T cells and their subtypes in pigs, we currently do not know the impact these cells may have in mounting a response to the i.u. vaccination.

To establish combining vaccines with semen during breeding as a viable alternative method of immunization, it is critical that we establish not only an effective immune response, but we also must ensure that sperm function and fertility are not negatively affected. Our results show that vaccinating gilts or sows via the i.u. route with recombinant proteins and/or inactivated PPV formulated with TriAdj did not negatively impact sperm function or motility, fetal viability, CR length or fetal weight suggesting that a properly formulated i.u. vaccine does not negatively impact fertility. Piglet weight at birth and weaning also did not appear to be negatively affected by i.u. vaccination. However, i.u. vaccines with inactivated virus or recombinant proteins did not promote a significant humoral response in gilts or sows when the i.u. vaccine was a primary immunization. Only sows that had previously been vaccinated with an i.m inactivated PPV vaccine produced a humoral anti-VP2 IgG, -IgG1, and -IgG2 immune response that was comparable to the i.m control sows. These results contrast with what has been observed in rats and rabbits which showed that a single i.u. vaccine triggered a measurable antigen-specific systemic and local humoral immunity (6–8). The reasons why the i.u. vaccine may have been effective in rodents or rats after a single dose may be due to the fact that they were administered without semen. Because we observed increased humoral immunity to a booster i.u. vaccine in sows that had previously received a primary systemic vaccine, it is possible for an i.u. vaccine to be effective under still undefined conditions. More trials need to be performed to clarify whether repeated i.u. vaccination can trigger strong humoral immunity or whether the primary response needs to occur via a systemic route. Of additional concern is the possibility of generating an immune response to sperm that results in infertility or reduced fertility in future pregnancies, as has been observed in in humans, mice and rabbits following immunization with sperm specific proteins (47). We hypothesize that by delivering the sperm through its conventional route, the mechanisms for prevention of infertility inducing immune responses to sperm will be maintained (41), however further studies will be required to determine if immunization utilizing an artificial insemination dose impacts future pregnancies. Lastly, we could establish whether the semen dose itself interferes with the efficacy of a primary immunization by administering the first i.u. dose in gilts during their first-heat detection.

## Data Availability Statement

All datasets generated for this study are included in the article/Supplementary Material.

## Ethics Statement

This animal study was reviewed and approved by University Animal Care Committee (UACC) University of Saskatchewan Animal Research Ethics Board (AREB).

## Author's Note

Portions of this manuscript are derived from the Ph.D. thesis of GH with permission from Dr. Janet Hill, the Department Head of Veterinary Microbiology, University of Saskatchewan. This manuscript was published with permission by the Director of VIDO-InterVac as journal series #893.

## Author Contributions

GH, JP, and HW conceived of and designed the experiments. GH carried out qPCR experiments, optimized and carried out the UEC isolation experiments as well as the laser-capture and stimulation experiments, analyzed all adjuvant alone data, and performed the CASA analysis. GH and JP developed and optimized all staining protocols, performed the vaccine trials, and processed the results. JP performed the flow cytometric analysis to measure sperm abnormality. JP, GH and SN performed the serum antibody analysis. KF, OS, and BD performed tissue processing and fetal measurements in the final trial. HW and GH drafted the manuscript. All authors read and approved the manuscript.

## Conflict of Interest

The authors declare that the research was conducted in the absence of any commercial or financial relationships that could be construed as a potential conflict of interest.

## Supplementary Material

Supplementary Figure 1 Schematic timeline of hormonal synchronization method for fixed time artificial insemination of sows.

Supplementary Figure 2 Gating strategy used for T cell, B cell, and monocyte immunotyping stains from the blood.

Supplementary Figure 3 Gating strategy used for myeloid cell immunotyping stain used for luminal cell populations.

Supplementary Figure 4 Changes in the primary uterine epithelial cell (UEC) transepithelial electrical resistance TEER stimulated with multiple adjuvant components alone and in combination. UECs were cultured until polarized and stimulated by adjuvant components (horizontal axis) and had the TEER measured prior to the addition of stimulants at 6 h **(A)** and again at 24 h **(B)**. Statistical analysis was done by Kruskal-Wallis test and significant differences between mock and individual stimulations were determined by Dunn's multiple comparison tests (^*^*p* < 0.05). Each circle, square, etc. represents a unique biological replicate and mean values are represented by a horizontal line.

Supplementary Figure 5 Representative immunohistofluorescence of CD163^+^ cells in uterine tissue after breeding with semen only (SO) or with a triple adjuvant combination (STA). **(A)** Twenty-four hours after breeding with semen alone or with TriAdj, uterine tissue was processed for immunohistofluorescence. Stained slides were imaged in 10 random fields of view and CD163 positive cells were counted by Image J **(B)** and significant differences were determined by unpaired *t*-test with Welch's correction. Each circle or square represents a unique biological replicate and the line represents mean data.

Supplementary Figure 6 Gene expression of uterine tissue and laser captured uterine epithelia (LC-UE) of sows 24 h following breeding with semen only (SO) or semen containing a triple adjuvant combination (STA). Gene expression analysis was performed for the following genes: TNFα, IFNβ, GM-CSF, IL6, IL8, CCL2, CCL3, CCL4, and CCL28. UTE expression shows averaged gene expression profiles across the lower, middle and upper uterine horn and LC-UE samples were collected from samples in the middle of the uterine horn. Significant differences within sample types were determined by unpaired *t*-test with Welch's correction (^*^*p* < 0.05). Each circle or square represents a unique biological replicate and the line represents mean data.

Supplementary Figure 7 Serum antibody titers from animals vaccinated through the i.u. or intramuscular routes (i.m.). Animals were bred with extended semen alone or with i.u. vaccine comprised of 1 × 10^7^ TCID_50_ BEI-inactivated PPV, 400 μg Poly I:C, 800 μg HDP, 400 μg PCEP (i.u. vaccine) and control sows (*n* = 3) were immunized with ParvoShield vaccine by i.m. route. All sows had previously been vaccinated i.m. with ParvoShield at each breeding cycle ~120 days previously. Serum anti-VP2 IgG **(A)**, IgG1 **(B)**, and IgG2 **(C)** antibody titres for i.u.-vaccinated (closed symbols) and i.m.-vaccinated (open symbols) sows. Percent change of serum anti-VP2 IgG **(D)**, IgG1 **(E)**, and IgG2 **(F)** antibody titres for i.u.-vaccinated (closed symbols) and i.m.-vaccinated (open symbols) sows are also shown.

Supplementary Figure 8 Weight of piglets born from IU-vaccinated and control gilts and anti-VP2 serum antibody titres over time. Intrauterine-vaccinated animals were bred with standard extended semen dose plus 800 μg recombinant VP2-Trx formulated with 400 μg Poly I:C, 800 μg HDP, and 400 μg PCEP. Control animals received the standard semen dose. Blood was obtained for the gilts day 0, 15, 30, 70, 90, and at wean (21 days after piglet birth). Piglet weights were measured on day 3 after birth **(A)** and at weaning **(B)** and the average weight of the piglets born to each gilt is shown. **(C)** Serum anti-VP2 IgG antibody titres were quantified relative to each gilt's anti-VP2 titres at day 0 to give relative anti-VP2 IgG titres for i.u.-vaccinated (orange circle) and i.m.-vaccinated (blue triangles) gilts. Horizontal bars present mean values.

Supplementary Table 1 Primer names, sequences, annealing temperature, and target sequence used in all qPCR experiments.

Supplementary Table 2 Antibodies used in FCM analysis, final concentrations, and suppliers.
