# Supplementary figures and images for: Assessment of Immunological Response and Impacts on Fertility Following Intrauterine Vaccination Delivered to Swine in an Artificial Insemination Dose
Source: Front Immunol. 2020 May 27;11:1015. doi: 10.3389/fimmu.2020.01015 (PMC7267065; doi:10.3389/fimmu.2020.01015)

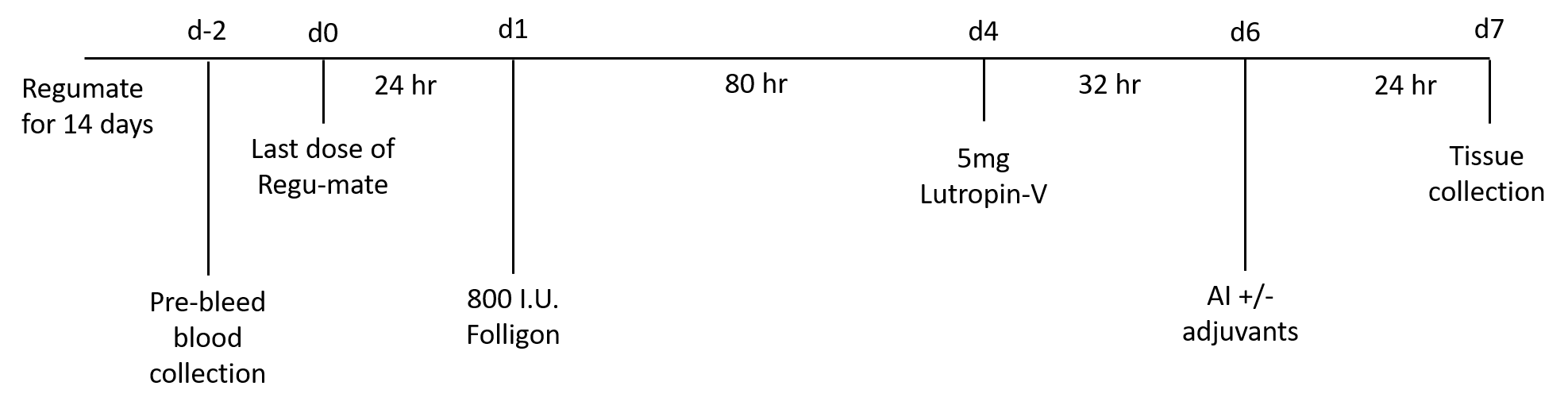

Supplement: Supplementary Figure 1 — Schematic timeline of hormonal synchronization method for fixed time artificial insemination of sows. [file Image_1.TIF]

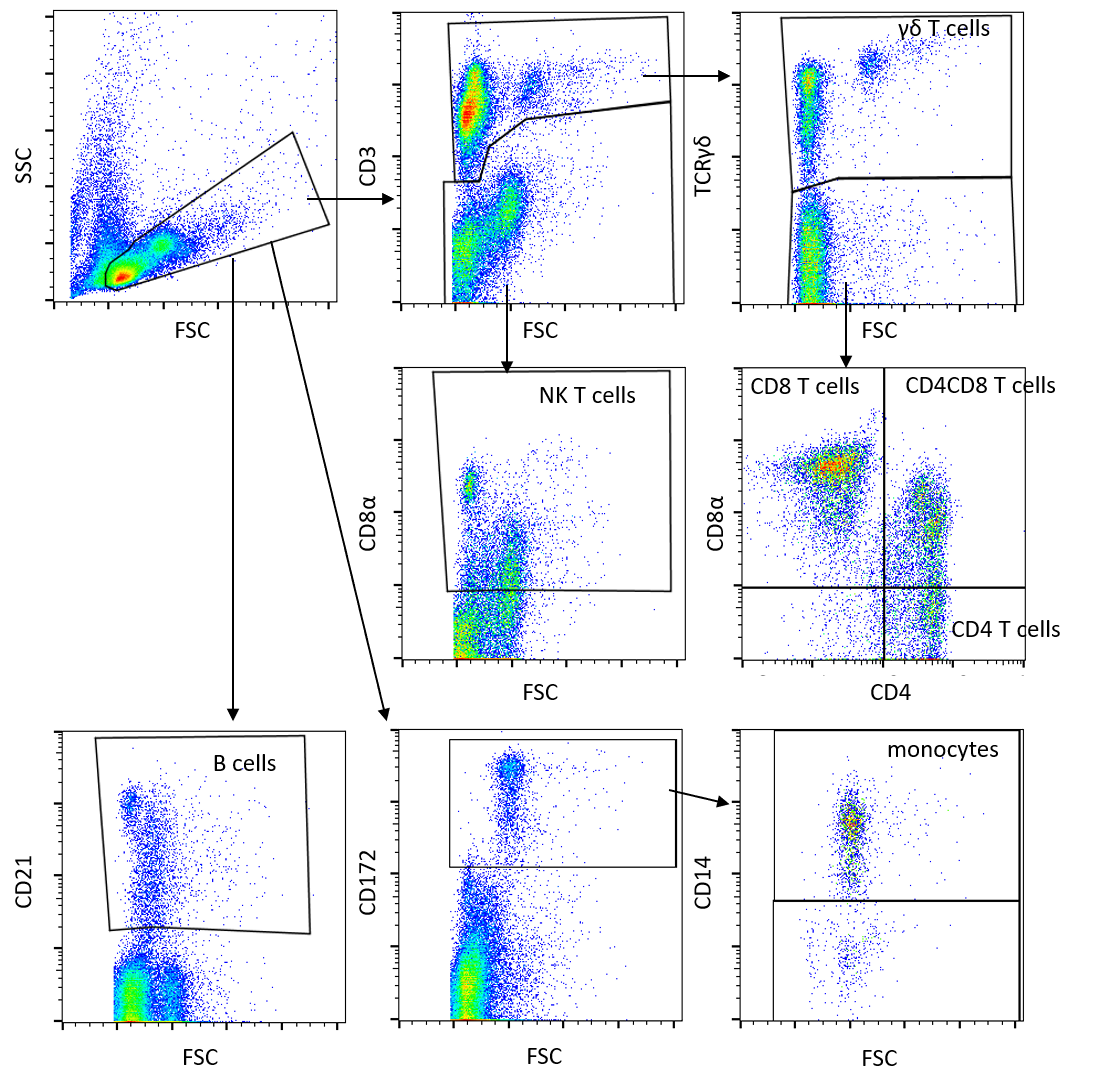

Supplement: Supplementary Figure 2 — Gating strategy used for T cell, B cell, and monocyte immunotyping stains from the blood. [file Image_2.TIF]

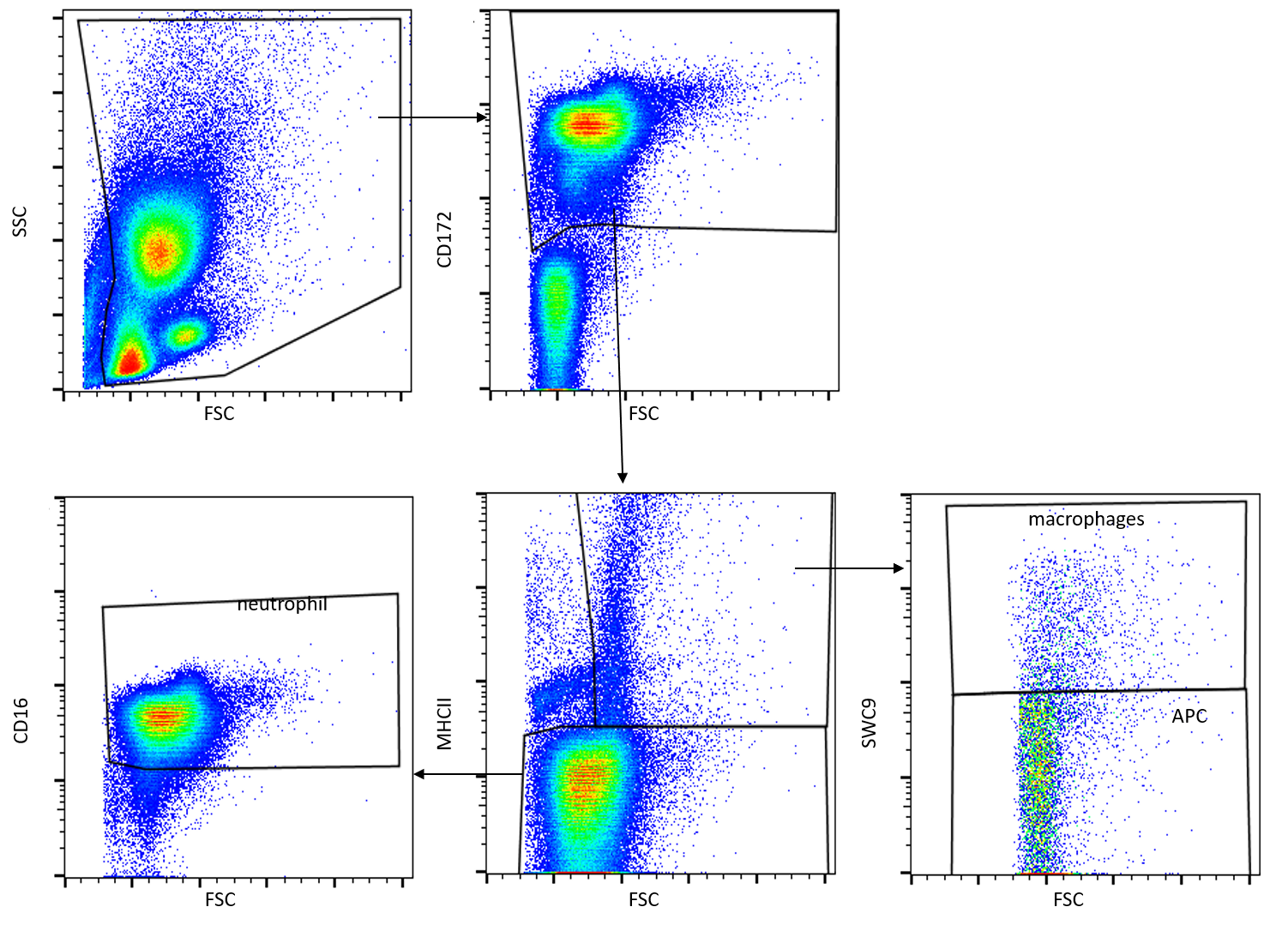

Supplement: Supplementary Figure 3 — Gating strategy used for myeloid cell immunotyping stain used for luminal cell populations. [file Image_3.TIF]

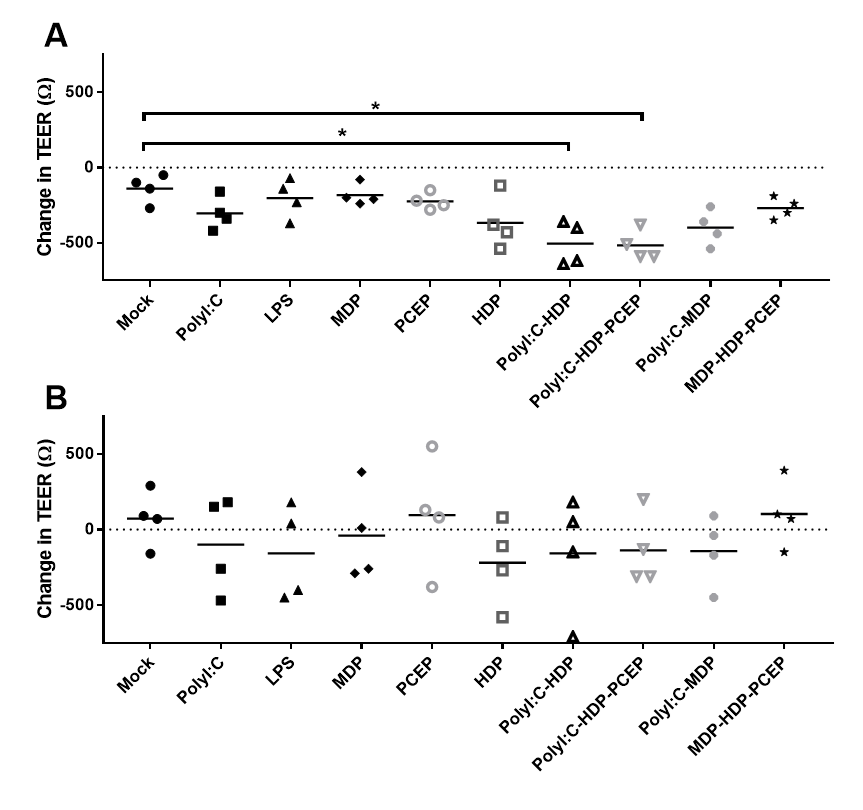

Supplement: Supplementary Figure 4 — Changes in the primary uterine epithelial cell (UEC) transepithelial electrical resistance TEER stimulated with multiple adjuvant components alone and in combination. UECs were cultured until polarized and stimulated by adjuvant components (horizontal axis) and had the TEER measured prior to the addition of stimulants at 6 h (A) and again at 24 h (B). Statistical analysis was done by Kruskal-Wallis test and significant differences between mock and individual stimulations were determined by Dunn's multiple comparison tests (*p < 0.05). Each circle, square, etc. represents a unique biological replicate and mean values are represented by a horizontal line. [file Image_4.TIF]

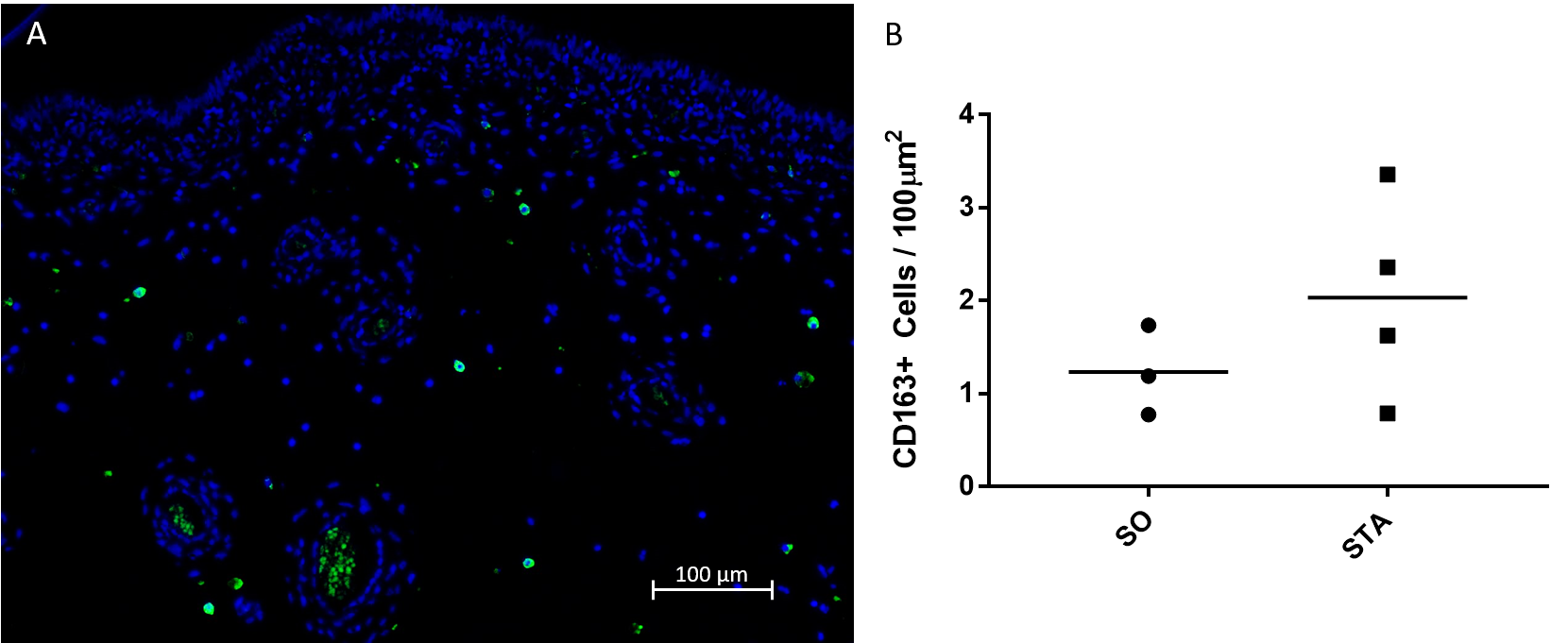

Supplement: Supplementary Figure 5 — Representative immunohistofluorescence of CD163+ cells in uterine tissue after breeding with semen only (SO) or with a triple adjuvant combination (STA). (A) Twenty-four hours after breeding with semen alone or with TriAdj, uterine tissue was processed for immunohistofluorescence. Stained slides were imaged in 10 random fields of view and CD163 positive cells were counted by Image J (B) and significant differences were determined by unpaired t-test with Welch's correction. Each circle or square represents a unique biological replicate and the line represents mean data. [file Image_5.TIF]

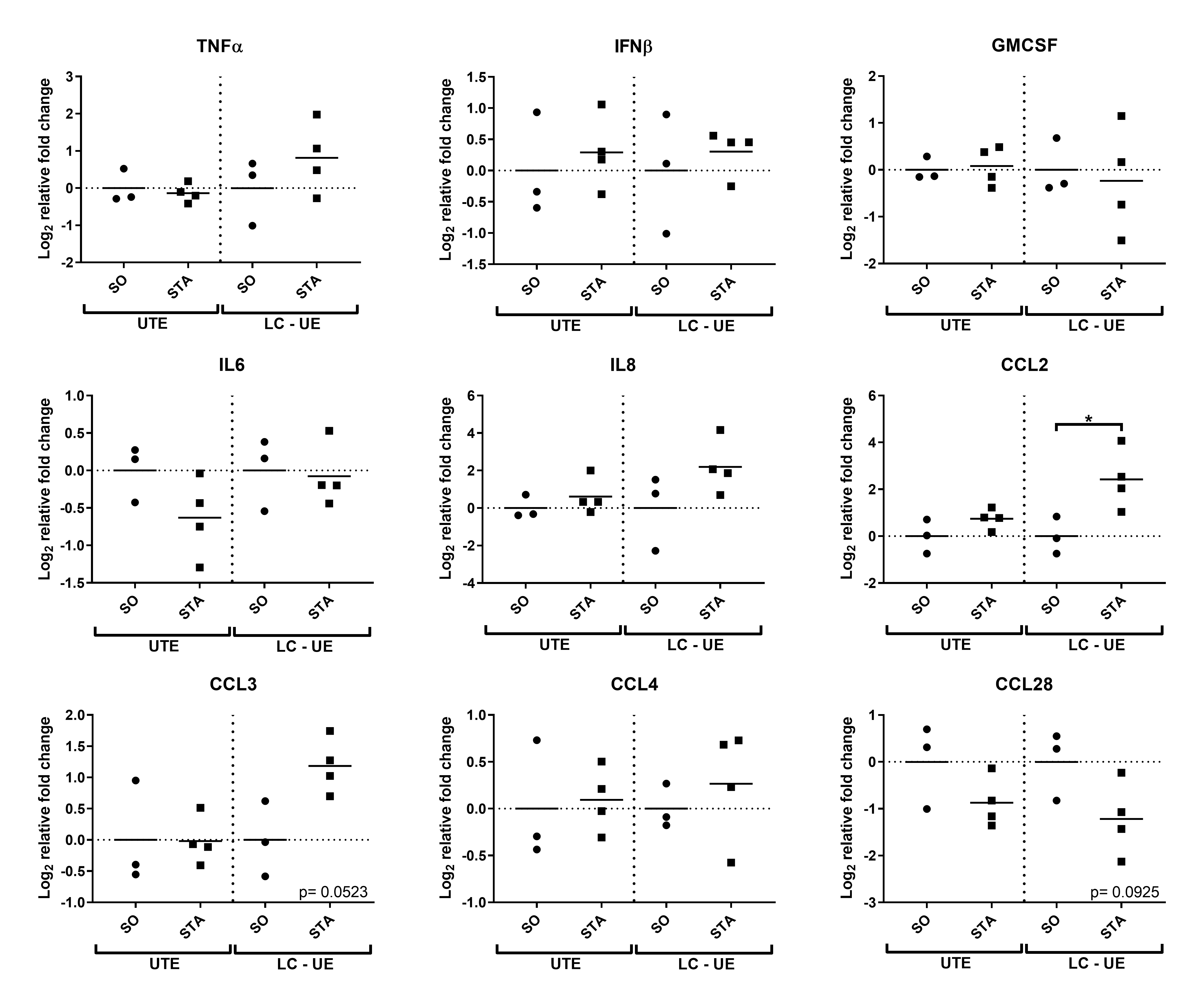

Supplement: Supplementary Figure 6 — Gene expression of uterine tissue and laser captured uterine epithelia (LC-UE) of sows 24 h following breeding with semen only (SO) or semen containing a triple adjuvant combination (STA). Gene expression analysis was performed for the following genes: TNFα, IFNβ, GM-CSF, IL6, IL8, CCL2, CCL3, CCL4, and CCL28. UTE expression shows averaged gene expression profiles across the lower, middle and upper uterine horn and LC-UE samples were collected from samples in the middle of the uterine horn. Significant differences within sample types were determined by unpaired t-test with Welch's correction (*p < 0.05). Each circle or square represents a unique biological replicate and the line represents mean data. [file Image_6.TIF]

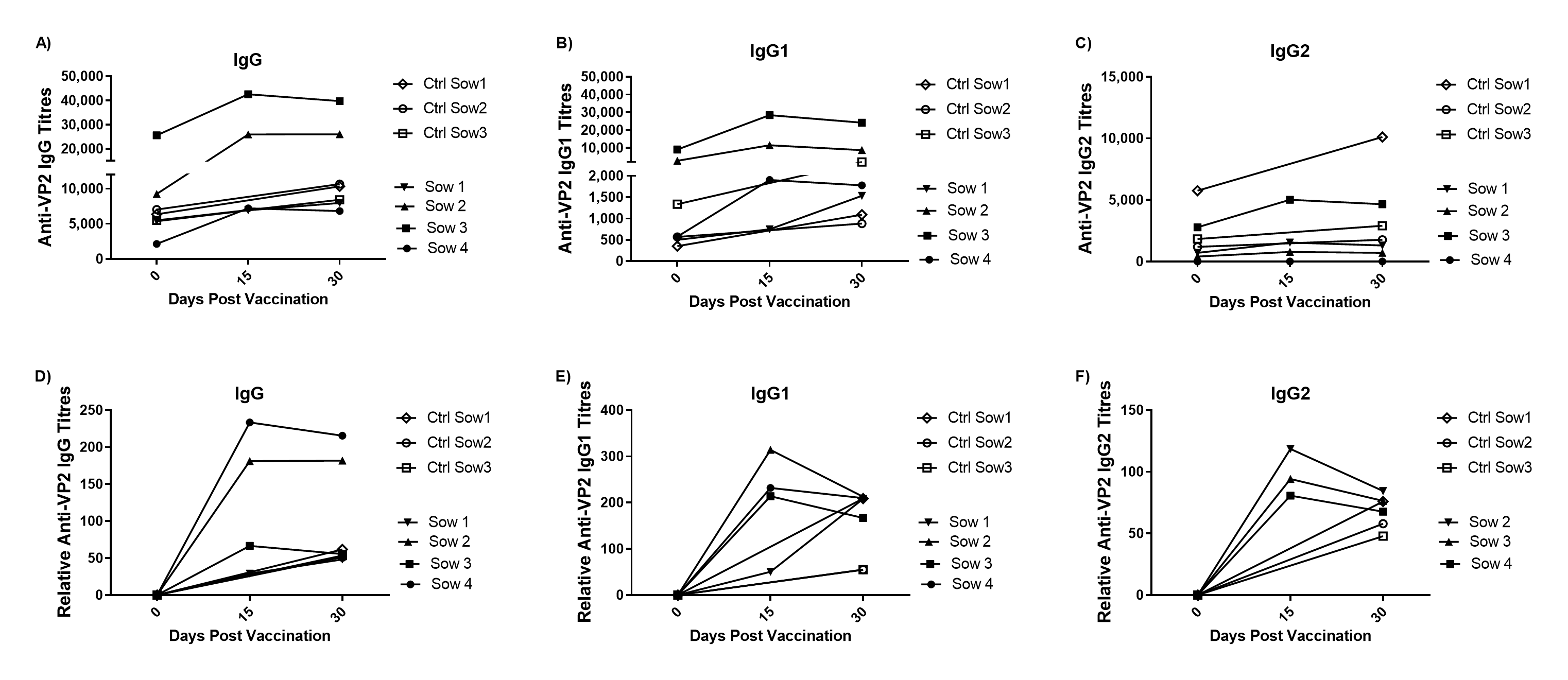

Supplement: Supplementary Figure 7 — Serum antibody titers from animals vaccinated through the i.u. or intramuscular routes (i.m.). Animals were bred with extended semen alone or with i.u. vaccine comprised of 1 × 10^7^ TCID50 BEI-inactivated PPV, 400 μg Poly I:C, 800 μg HDP, 400 μg PCEP (i.u. vaccine) and control sows (n = 3) were immunized with ParvoShield vaccine by i.m. route. All sows had previously been vaccinated i.m. with ParvoShield at each breeding cycle ~120 days previously. Serum anti-VP2 IgG (A), IgG1 (B), and IgG2 (C) antibody titres for i.u.-vaccinated (closed symbols) and i.m.-vaccinated (open symbols) sows. Percent change of serum anti-VP2 IgG (D), IgG1 (E), and IgG2 (F) antibody titres for i.u.-vaccinated (closed symbols) and i.m.-vaccinated (open symbols) sows are also shown. [file Image_7.tif]

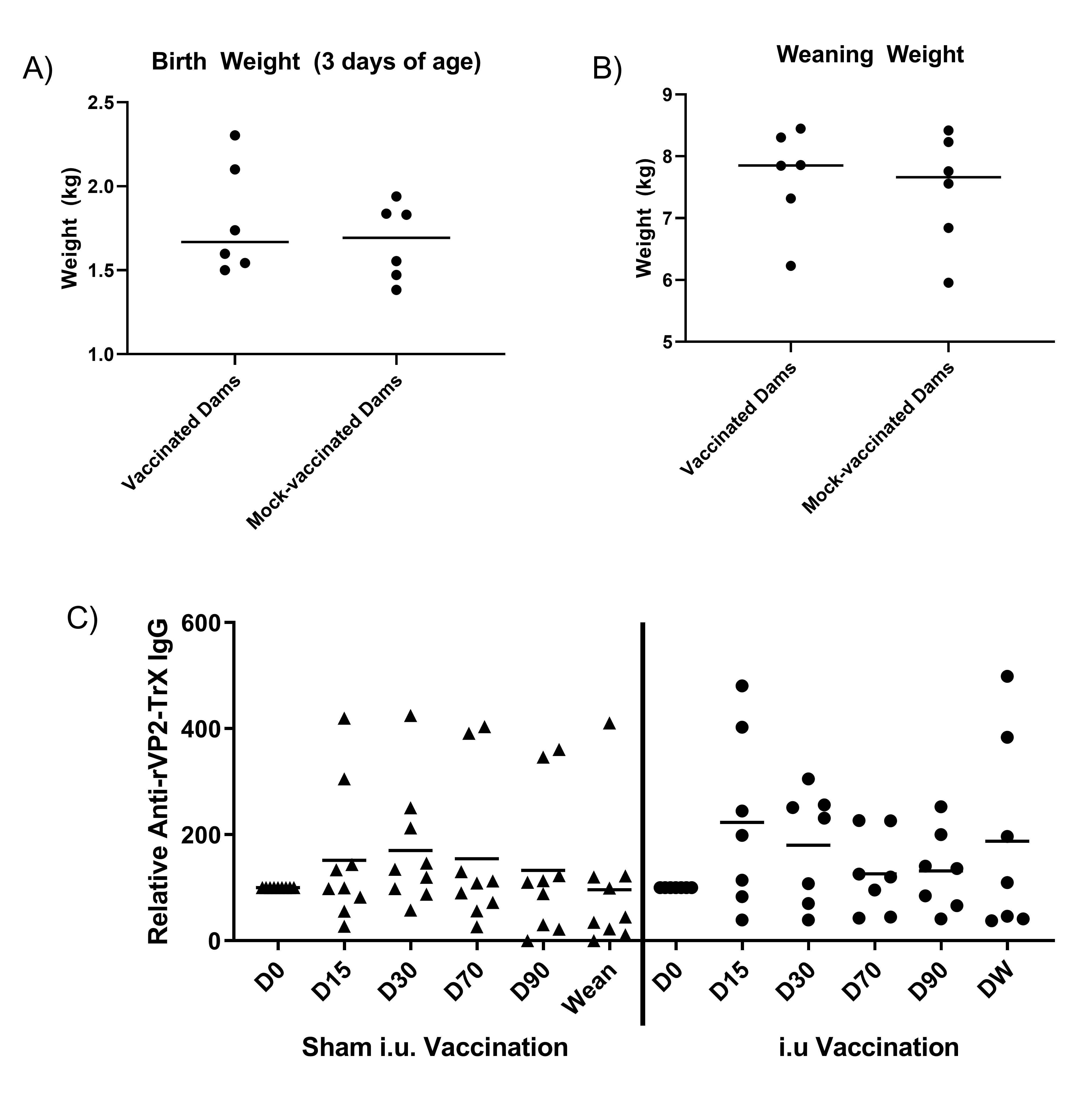

Supplement: Supplementary Figure 8 — Weight of piglets born from IU-vaccinated and control gilts and anti-VP2 serum antibody titres over time. Intrauterine-vaccinated animals were bred with standard extended semen dose plus 800 μg recombinant VP2-Trx formulated with 400 μg Poly I:C, 800 μg HDP, and 400 μg PCEP. Control animals received the standard semen dose. Blood was obtained for the gilts day 0, 15, 30, 70, 90, and at wean (21 days after piglet birth). Piglet weights were measured on day 3 after birth (A) and at weaning (B) and the average weight of the piglets born to each gilt is shown. (C) Serum anti-VP2 IgG antibody titres were quantified relative to each gilt's anti-VP2 titres at day 0 to give relative anti-VP2 IgG titres for i.u.-vaccinated (orange circle) and i.m.-vaccinated (blue triangles) gilts. Horizontal bars present mean values. [file Image_8.tif]
