# Supplementary material for: Assessment of Immunological Response and Impacts on Fertility Following Intrauterine Vaccination Delivered to Swine in an Artificial Insemination Dose
Source: Front Immunol. 2020 May 27;11:1015. doi: 10.3389/fimmu.2020.01015 (PMC7267065; doi:10.3389/fimmu.2020.01015)
Supplement: Supplementary Table 1 — Primer names, sequences, annealing temperature, and target sequence used in all qPCR experiments. [file Table_1.docx]

Supplementary Table 1. Primer names, sequences, annealing temperature and target sequence used in all qPCR experiments.

| Gene Name | Forward primer (5’-3’) | Reverse Primer (5’-3’) | Tm (°C) | Target sequence or reference |
| --- | --- | --- | --- | --- |
| RPL19 | AACTCCCGTCAGCAGATCC | AGTACCCTTCCGCTTACCG | 60 | [45] |
| YWHAZ | TGATGATAAGAAAGGGATTGTGG | GTTCAGCAATGGCTTCATCA | 60 | [46] |
| GAPDH | CTTCACGACCATGGAGAAGG | CCAAGCAGTTGGTGGTACAG | 63 | [47] |
| ActB | CACGCCATCCTGCGTCTGGA | AGCACCGTGTTGGCGTAGAG | 63 | [46] |
| IFNβ | AGTTGCCTGGGACTCCTCAA | CCTCAGGGACCTCAAAGTTCAT | 60 | [48] |
| TNFα | CCAATGGCAGAGTGGGTATG | TGAAGAGGACCTGGGAGTAG | 60 | [49] |
| GMCSF | GAAACCGTAGACGTCGTCTG | GTGCTGCTCATAGTGCTTGG | 62 | [49] |
| IL6 | ATCAGGAGACCTGCTTGATG | TGGTGGCTTTGTCTGGATTC | 60 | [49] |
| IL8 | TCCTGCTTTCTGCAGCTCTC | GGGTGGAAAGGTGTGGAATG | 62 | [49] |
| CCL2 | AGTCACCTGCTGCTATACAC | GCGATGGTCTTGAAGATCAC | 60 | NM_214214 |
| CCL3 | GCCTGCTGCTTCTCCTATAC | TCAGCTCCAGGTCAGAGATG | 60 | AY643423 |
| CCL4 | AACCTCTCCTCCAGCAAGAC | GTCACGAAGTTGCGAGGAAG | 60 | NM_213779 |
| CCL20 | GCTCCTGGCTGCTTTGATGTC | CATTGGCGAGCTGCTGTGTG | 66 | [49] |
| CCL28 | GCTGCTGCACTGAGGTTTC | TGAGGGCTGACACAGATTC | 62 | [49] |
| SLA-DRA | ATCTCCCCTTCATGCCCTCA | AGCTTCAAACTCCCAGTGCT | 60 | NM_001113706 |
