# Supplementary material for: Assessment of Immunological Response and Impacts on Fertility Following Intrauterine Vaccination Delivered to Swine in an Artificial Insemination Dose
Source: Front Immunol. 2020 May 27;11:1015. doi: 10.3389/fimmu.2020.01015 (PMC7267065; doi:10.3389/fimmu.2020.01015)
Supplement: Supplementary Table 2 — Antibodies used in FCM analysis, final concentrations, and suppliers. [file Table_2.docx]

Supplementary Table 2. Antibodies used in FCM analysis, final concentrations, and suppliers.

| Target | Clone (Isotype) | Fluorochrome | Final concentration | Supplier |
| --- | --- | --- | --- | --- |
| Primary Antibody targets | | | | |
| Anti-CD4 | 74-12-4 (mIgG2b) | N/A | 10 µg/ml | Monoclonal Antibody Center |
| Anti-CD8α | 76-2-11 (mIgG2a) | N/A | 10 µg/ml | Monoclonal Antibody Center |
| Anti-TCRγδ | PGBL22A (mIgG1) | N/A | 5 µg/ml | Kingfisher Biotech |
| Anti-CD3 | PPT3 (mIgG1) | PE | 10 µg/ml | Southern Biotech |
| Anti-CD21 | BB6-11C9.6 (mIgG1) | N/A | 5 µg/ml | Southern Biotech |
| Anti-CD14 | Cam36A (mIgG1) | N/A | 5 µg/ml | Monoclonal Antibody Center |
| Anti-CD172 | 74-22-15A (mIgG2b) | N/A | 2 µg/ml | Monoclonal Antibody Center |
| Anti-MHCII | MSA3 (mIgG2a) | N/A | 2 µg/ml | Kingfisher Biotech |
| Anti-SWC9 | PM18-7(mIgG1) | Biotin | 2 µg/ml | Bio-Rad Antibodies |
| Anti-CD16 | G7 (mIgG1) | Cye5 | 5 µg/ml | Bio-Rad Antibodies |
| Secondary Antibody targets | | | | |
| N/A | IgG | N/A | 10 µg/ml | Chrompure |
| Anti-mIgG1 | N/A | Biotin | 1.25 µg/ml | Southern Biotech |
| Anti-mIgG1 | N/A | APC | 1.25 µg/ml | Southern Biotech |
| Anti-mIgG1 | N/A | PE | 1.25 µg/ml | Southern Biotech |
| Anti-mIgG2a | N/A | Alexa647 | 2.5 µg/ml | Southern Biotech |
| Anti-mIgG2a | N/A | PE | 1.25 µg/ml | Southern Biotech |
| Anti-mIgG2b | N/A | FITC | 1.25 µg/ml | Southern Biotech |
| Anti-mIgG2b | N/A | APC | 1.25 µg/ml | Southern Biotech |
| Streptavidin | N/A | PerCP-Cy5.5 | 0.5 µg/ml | eBioscience |
